# Supplementary figures and images for: Metarhizium spp. isolates effective against Queensland fruit fly juvenile life stages in soil
Source: PLoS One. 2024 Jan 18;19(1):e0297341. doi: 10.1371/journal.pone.0297341 (PMC10796031; doi:10.1371/journal.pone.0297341)

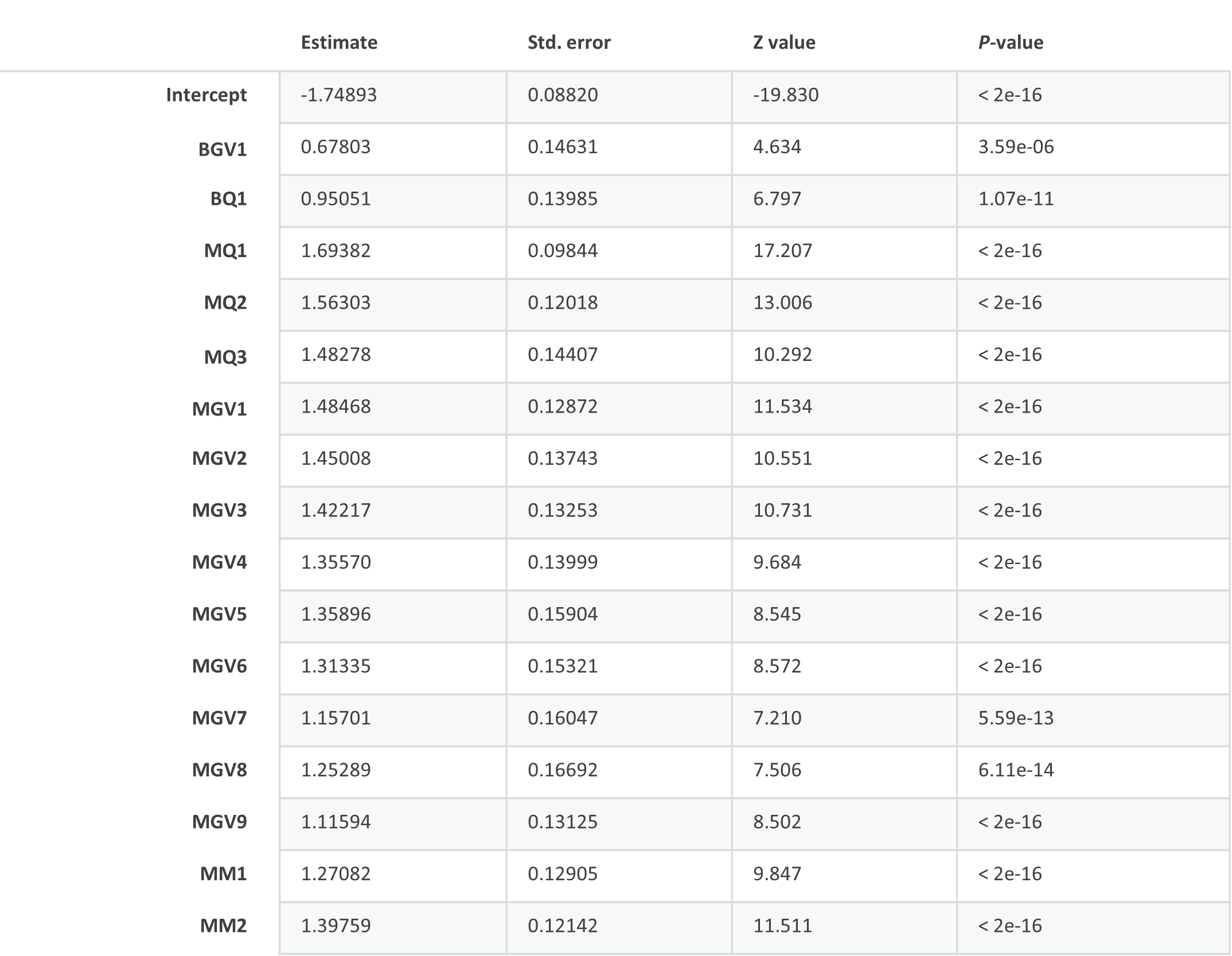

Supplement: S1 Table — The estimated variance for the random effect ‘bioassay’ is 0.016. (TIF) [file pone.0297341.s002.tif]
