# Supplementary material for: Metarhizium spp. isolates effective against Queensland fruit fly juvenile life stages in soil
Source: PLoS One. 2024 Jan 18;19(1):e0297341. doi: 10.1371/journal.pone.0297341 (PMC10796031; doi:10.1371/journal.pone.0297341)
Supplement: Attachment — Submitted filename: PONE-D-23-37553.pdf [file pone.0297341.s004.pdf]

PLOS ONE

Metarhizium

spp.

isolates effective against Queensland fruit fly juvenile life stages in soil.

--Manuscript Draft--

Manuscript Number:	PONE-D-23-37553
Article Type:	Research Article
Full Title:	Metarhizium<br><br>spp.<br><br>isolates effective against Queensland fruit fly juvenile life stages in soil.
Short Title:	Metarhizium spp. isolates effective against Queensland fruit fly juvenile life stages in soil.
Corresponding Author:	Madita Prince<br>Agriculture Victoria<br>Tatura, Victoria AUSTRALIA
Keywords:	Queensland Fruit Fly, Bactrocera tryoni, Tephritidae, Integrated Pest Management, Metarhizium, Beauveria, Entomopathogenic Fungi
Abstract:	Queensland fruit fly, Bactrocera tryoni, Froggatt (Diptera: Tephritidae) is Australia’s primary fruit fly pest species. Integrated Pest Management (IPM) has been adopted to sustainably manage this polyphagous species with a reduced reliance on chemical pesticides. At present, control measures are aimed at the adult stages of the fly, with no IPM tools available to target larvae once they exit the fruit and pupate in the soil. The use of entomopathogenic fungi may provide a biologically-based control method for these soil-dwelling life stages. The effectiveness of Metarhizium and Beauveria species fungal isolates were screened under laboratory conditions against Queensland fruit fly. In bioassays, 16 isolates were screened for pathogenicity following exposure of third-instar larvae to inoculum-treated vermiculite used as a pupation substrate. The best performing Metarhizium sp. isolate achieved an average percentage mortality of 93 %, whereas the best performing Beauveria isolate was less efficient, with an average mortality of 36 %. Susceptibility to infection during different development stages was investigated using selected fungal isolates, with the aim of assessing all soil-dwelling life stages from third-instar larvae to final pupal stages and emerging adults. Overall, the third larval instar was the most susceptible stage, with average mortalities between 51 - 98 % depending on the isolate tested. Moreover, adult mortality was significantly higher when exposed to inoculum during pupal eclosion, with mortalities between 56 - 76 % observed within the first nine days post-emergence. The effect of temperature and inoculum concentration on insect mortality were assessed independently with candidate isolates to determine the optimum temperature range for fungal biological control activity and the rate required for application in field conditions. Metarhizium spp. are highly efficacious at killing Queensland fruit fly and have potential for use as biopesticides to target soil-dwelling and other life stages of B. tryoni.
Order of Authors:	Madita Prince
	Aimee C McKinnon
	Diana Leemon
	Tim I Sawbridge
	John Paul Cunningham
Additional Information:	
Question	Response

Powered by Editorial Manager® and ProduXion Manager® from Aries Systems Corporation

**Financial Disclosure**<br><br>Enter a financial disclosure statement that describes the sources of funding for the work included in this submission. Review the submission guidelines for detailed requirements. View published research articles from *PLOS ONE* for specific examples.<br><br>This statement is required for submission and **will appear in the published article** if the submission is accepted. Please make sure it is accurate.<br><br>**Funded studies**<br>Enter a statement with the following details:<br>• Initials of the authors who received each award<br>• Grant numbers awarded to each author<br>• The full name of each funder<br>• URL of each funder website<br>• Did the sponsors or funders play any role in the study design, data collection and analysis, decision to publish, or preparation of the manuscript?<br><br>Did you receive funding for this work?	Yes
Please add funding details.<br>as follow-up to "**Financial Disclosure**<br><br>Enter a financial disclosure statement that describes the sources of funding for the work included in this submission. Review the submission guidelines for detailed requirements. View published research articles from *PLOS ONE* for specific examples.<br><br>This statement is required for submission and **will appear in the published article** if the submission is accepted. Please make sure it is accurate.	MP: This research was conducted as part of a PhD fellowship with financial support from Agriculture Victoria (https://agriculture.vic.gov.au/) and La Trobe University (https://www.latrobe.edu.au).<br><br>Work was carried out under the project: A national biocontrol program to manage pest fruit flies in Australia (Project ID: 4-EKSH327)

Powered by Editorial Manager® and ProduXion Manager® from Aries Systems Corporation

**Funded studies**<br>Enter a statement with the following details:<br>• Initials of the authors who received each award<br>• Grant numbers awarded to each author<br>• The full name of each funder<br>• URL of each funder website<br>• Did the sponsors or funders play any role in the study design, data collection and analysis, decision to publish, or preparation of the manuscript?<br><br>Did you receive funding for this work?"	
Please select the country of your main research funder (please select carefully as in some cases this is used in fee calculation).<br>as follow-up to "**Financial Disclosure**<br><br>Enter a financial disclosure statement that describes the sources of funding for the work included in this submission. Review the submission guidelines for detailed requirements. View published research articles from *PLOS ONE* for specific examples.<br><br>This statement is required for submission and **will appear in the published article** if the submission is accepted. Please make sure it is accurate.<br><br>**Funded studies**<br>Enter a statement with the following details:<br>• Initials of the authors who received each award<br>• Grant numbers awarded to each author<br>• The full name of each funder<br>• URL of each funder website<br>• Did the sponsors or funders play any role in the study design, data collection and analysis, decision to publish, or preparation of the manuscript?<br><br>Did you receive funding for this work?"	AUSTRALIA - AU
**Competing Interests**	The authors have declared that no competing interests exist.

Use the instructions below to enter a competing interest statement for this submission. On behalf of all authors, disclose any competing interests that could be perceived to bias this work—acknowledging all financial support and any other relevant financial or non-financial competing interests.<br><br>This statement is required for submission and **will appear in the published article** if the submission is accepted. Please make sure it is accurate and that any funding sources listed in your Funding Information later in the submission form are also declared in your Financial Disclosure statement.<br><br>View published research articles from *PLOS ONE* for specific examples.<br><br>**NO authors have competing interests**<br><br>Enter: *The authors have declared that no competing interests exist.*<br><br>**Authors with competing interests**<br><br>Enter competing interest details beginning with this statement:<br><br>*I have read the journal's policy and the authors of this manuscript have the following competing interests: [insert competing interests here]*<br><br>* typeset	
**Ethics Statement**<br><br>Enter an ethics statement for this submission. This statement is required if the study involved:<br><br>• Human participants<br>• Human specimens or tissue<br>• Vertebrate animals or cephalopods<br>• Vertebrate embryos or tissues	N/A

Powered by Editorial Manager® and ProduXion Manager® from Aries Systems Corporation

• Field research<br><br>Write "N/A" if the submission does not require an ethics statement.<br><br>General guidance is provided below. Consult the submission guidelines for detailed instructions. **Make sure that all information entered here is included in the Methods section of the manuscript.**	

**Format for specific study types**<br><br>**Human Subject Research (involving human participants and/or tissue)**<br>• Give the name of the institutional review board or ethics committee that approved the study<br>• Include the approval number and/or a statement indicating approval of this research<br>• Indicate the form of consent obtained (written/oral) or the reason that consent was not obtained (e.g. the data were analyzed anonymously)<br><br>**Animal Research (involving vertebrate animals, embryos or tissues)**<br>• Provide the name of the Institutional Animal Care and Use Committee (IACUC) or other relevant ethics board that reviewed the study protocol, and indicate whether they approved this research or granted a formal waiver of ethical approval<br>• Include an approval number if one was obtained<br>• If the study involved *non-human primates*, add *additional details* about animal welfare and steps taken to ameliorate suffering<br>• If anesthesia, euthanasia, or any kind of animal sacrifice is part of the study, include briefly which substances and/or methods were applied<br><br>**Field Research**<br><br>Include the following details if this study involves the collection of plant, animal, or other materials from a natural setting:<br>• Field permit number<br>• Name of the institution or relevant body that granted permission	
**Data Availability**<br><br>Authors are required to make all data underlying the findings described fully available, without restriction, and from the time of publication. PLOS allows rare exceptions to address legal and ethical concerns. See the PLOS Data Policy and FAQ for detailed information.	No - some restrictions will apply

A Data Availability Statement describing where the data can be found is required at submission. Your answers to this question constitute the Data Availability Statement and **will be published in the article**, if accepted.<br><br>**Important:** Stating 'data available on request from the author' is not sufficient. If your data are only available upon request, select 'No' for the first question and explain your exceptional situation in the text box.<br><br>Do the authors confirm that all data underlying the findings described in their manuscript are fully available without restriction?	
**Describe where the data may be found in full sentences. If you are copying our sample text, replace any instances of XXX with the appropriate details.**<br><br>• If the data are **held or will be held in a public repository**, include URLs, accession numbers or DOIs. If this information will only be available after acceptance, indicate this by ticking the box below. For example: *All XXX files are available from the XXX database (accession number(s) XXX, XXX.).*<br>• If the data are all contained **within the manuscript and/or Supporting Information files**, enter the following: *All relevant data are within the manuscript and its Supporting Information files.*<br>• If neither of these applies but you are able to provide **details of access elsewhere**, with or without limitations, please do so. For example:<br><br>*Data cannot be shared publicly because of [XXX]. Data are available from the XXX Institutional Data Access / Ethics Committee (contact via XXX) for researchers who meet the criteria for access to confidential data.*<br><br>*The data underlying the results presented in the study are available from (include the name of the third party*	Data available on request from the author. Data cannot be shared publicly because data availability restrictions applies.

and contact information or URL).<br>• This text is appropriate if the data are owned by a third party and authors do not have permission to share the data.<br><br>* typeset	
Additional data availability information:	Tick here if your circumstances are not covered by the questions above and you need the journal’s help to make your data available.; Tick here if the URLs/accession numbers/DOIs will be available only after acceptance of the manuscript for publication so that we can ensure their inclusion before publication.

Powered by Editorial Manager® and ProduXion Manager® from Aries Systems Corporation

Click here to access/download;Manuscript;Manuscript.docx

Metarhizium spp. isolates effective against Queensland fruit fly juvenile life stages in soil.

Madita Prince[1*], Aimee C. McKinnon[2], Diana Leemon[3], Tim Sawbridge[2,4], John Paul Cunningham[2,4]

[1] Agriculture Victoria, Tatura SmartFarm, Tatura, VIC, Australia

[2] Agriculture Victoria, Agribio Centre for AgriBiosciences, Bundoora, VIC, Australia

[3] Myco-Vation, Bellbowrie, QLD, Australia

[4] School of Applied Systems Biology, La Trobe University, Melbourne, VIC, Australia

[*] Corresponding author

E-mail: madita.prince@ecodev.vic.gov.au

Abstract

Queensland fruit fly, *Bactrocera tryoni*, Froggatt (Diptera: Tephritidae) is Australia's primary fruit fly pest species. Integrated Pest Management (IPM) has been adopted to sustainably manage this polyphagous species with a reduced reliance on chemical pesticides. At present, control measures are aimed at the adult stages of the fly, with no IPM tools available to target larvae once they exit the fruit and pupate in the soil. The use of entomopathogenic fungi may provide a biologically-based control method for these soil-dwelling life stages. The effectiveness of *Metarhizium* and *Beauveria* species fungal isolates were screened under laboratory conditions against Queensland fruit fly. In bioassays, 16 isolates were screened for pathogenicity following exposure of third-instar larvae to inoculum-treated vermiculite used as a pupation substrate. The best performing *Metarhizium* sp. isolate achieved an average percentage mortality of 93 %, whereas the best performing *Beauveria* isolate was less efficient, with an average mortality of 36 %. Susceptibility to infection during different development stages was investigated using selected fungal isolates, with the aim of assessing all soil-dwelling life stages from third-instar larvae to final pupal stages and emerging adults. Overall, the third larval instar was the most susceptible stage, with average mortalities between 51 - 98 % depending on the isolate tested. Moreover, adult mortality was significantly higher when exposed to inoculum during pupal eclosion, with mortalities between 56 - 76 % observed within the first nine days post-emergence. The effect of temperature and inoculum concentration on insect mortality were assessed independently with candidate isolates to determine the optimum temperature range for fungal biological control activity and the rate required for application in field conditions. *Metarhizium spp.* are highly efficacious at killing Queensland fruit fly and have potential for use as biopesticides to target soil-dwelling and other life stages of *B. tryoni.*

Introduction

Tephritid fruit flies are becoming increasingly problematic ~~to~~ Australia's $15 billion horticultural industry [1-3]. The Queensland fruit fly, *Bactrocera tryoni* Froggatt (Diptera: Tephritidae), is the primary tephritid pest; a highly polyphagous pest that is native to the country and causes considerable losses to production and market access [2, 4]. Queensland fruit fly is endemic to tropical and subtropical areas, but is now established widely throughout eastern Australia, having extended its geographical distribution into more temperate regions of southern Australia [3]. In Victoria, changes in climate over the past decade may be providing habitats that are more suitable for Queensland fruit fly populations, and the local climate is projected to become increasingly suited over time [1, 5].

The last decade has seen restricted use and withdrawal of several chemical insecticides that were previously relied upon for controlling fruit fly pests in Australia [6, 7]. Widespread pesticide use has been replaced by sustainable tools and practices that include protein bait spraying, male annihilation technique (MAT), female mass trapping, and the potential for sterile insect technique (SIT) [8-10]. These strategies target adult flies in the population, and flies that evade these control techniques may continue to reproduce, enabling local populations to persist. A more holistic strategy that targets all life stages of the fly may be more effective [11, 12]. The use of parasitoid wasp species that specifically target the egg and early larval stages of Queensland fruit fly developing in the fruit has been explored as augmentative (mass release) and conservation biological control (habitat improvement) approaches [13, 14]. Good orchard hygiene practices (i.e., removing fallen fruit) can also help reduce late-stage larvae that have escaped parasitism, but once they exit the fruit to pupate in soil, they can complete their development.

Entomopathogenic fungi are microorganisms that can be applied as biological control agents in liquid solutions, as contact powder, or as a bait formulation to infect and kill arthropod pests [15, 16]. Two entomopathogenic fungal species of the order Hypocreales, *Metarhizium anisopliae*

complex (Metschn.) Sorokin (family: Clavicipitaceae) and *Beauveria bassiana* (Bals.-Criv.) Vuill. (family: Cordycipitaceae) have had the most attention as potential biocontrol agents for tephritid fruit fly control, [17]. These entomopathogens are known for their safety [18, 19], ease of mass production [20] and pathogenicity against diverse insect groups, including tephritid fruit flies [21-25]. *M. anisopliae* and *B. bassiana* has been widely researched to manage South American fruit fly (*Anastrepha fraterculus*) [26], Mexican fruit fly (*A. ludens*) [26-29], Mediterranean fruit fly (*Ceratitis capitata*) [23, 30-33], mango fruit fly (*C. cosyra*) [31, 32], olive fruit fly (*Bactrocera oleae*) [34], Oriental fruit fly (*B. dorsalis*) [35, 36], carambola fruit fly (*B. carambolae*) [37, 38], and peach fruit fly (*B. zonata*) [35, 36, 39, 40]. These two entomopathogenic fungi species have been shown to exert pathogenicity against all life stages (larvae, pupae, and adult life stage) of the Mediterranean fruit fly (*C. capitata*) under laboratory conditions [23, 30, 33]. Exposure of pupating larvae of Oriental fruit fly (*B. dorsalis*) and olive fruit fly (*B. oleae*) to *M. anisopliae*- or *M. brunneum*-inoculated soil resulted in reduced adult emergence of both flies [11, 34]. When adult and juvenile life stages of Oriental fruit fly (*B. dorsalis*) and peach fruit fly (*B. zonata*) where tested, the same fungal isolates were highly effective as well [35, 40]. There have been no studies to date reporting on the effectiveness of entomopathogenic fungi against juvenile life stages of Queensland fruit fly, and only one study that reports on the efficacy of a single isolate of *M. anisopliae* against adult Queensland fruit fly [24].

We explored the potential for entomopathogenic fungi to be used as a soil application to control juvenile life stages of Queensland fruit fly. Focussing on *Beauveria bassiana* and *Metarhizium anisopliae sensu lato* (*M. anisopliae* s.l.) species, fungal isolates were obtained from soil collected from the Australian states of Queensland (eastern) and Victoria (southern). Isolates were screened in bioassays for their pathogenicity against late-stage larvae, pupae and emerging adults. We developed a bioassay to screen fungal entomopathogenic species and isolates, assessing host mortality rates under laboratory conditions [41]. To improve the chance of future field efficiency of the prospective isolates, we screened the suitability of isolates through testing the effect of

temperature on the isolate growth attributes [16, 42, 43], in addition to their pathogenicity against Queensland fruit fly. ~~Virulence~~ of an isolate likely differs from more sterile conditions (vermiculite) through interactions between the inoculum and resident soil microbiota [44, 45]. To explore this further, the most pathogenic isolate tested was evaluated in non-sterile orchard soil against third-instar larvae.

Methodology

Insects

A laboratory culture of Queensland fruit fly was maintained at the Tatura SmartFarm, Victoria, Australia, at 25 °C, 60 % relative humidity (RH), and 16:8 (L:D) photoperiod. Adult flies were housed in fine mesh insect cages (30 cm x 30 cm x 30 cm) where they were provided with water and a mixture of sugar and hydrolysed enzymatic yeast (3:1) as a food substrate [46]. A perforated plastic cup (30 mL with 14 holes 1.1 mm in diameter) lined with apple juice-soaked filter paper (no. 1) was placed for four hours in the cage~~,~~ for mated female flies to oviposit into. Eggs laid on the filter paper were placed onto artificial carrot-based larval diet (dehydrated carrot (20%), brewer's yeast (6.7 %), and methyl 4-hydroxybenzoate (0.67 %) per ~~litre~~ sdH_2O) in containers (500 mL), with approximately 30 eggs per 15 g of diet. The containers were placed in a second container (7.5 l) filled with 2 cm of vermiculite and secured with a lid. Larvae were left to feed on the diet until they reached the late third-instar "popping" stage, at which point they left the diet to pupate in provided vermiculite.

Isolation and identification of entomopathogenic fungi

Fungal entomopathogens were isolated from soil collected from ~~an~~ biodynamic orchard located in the Goulburn Valley region of Victoria, Australia (April 2021) using (i) serial dilution plating on selective media and (ii) insect-bait method [47]. 12 soil samples were collected across the sampling

site (1.25 ha) in a depth of 10-15 cm, using a manual core sampler. Each sample consisted of 20 subsamples (approximately 120 g soil) and was transferred into polyethylene bags, sealed, and stored in a portable cooler in the field. The sampler was sterilised (80 % ethanol) after each collection to avoid cross-contamination. All samples were transported to the laboratory and stored at 4 °C for further processing. For easier handling the samples were sieved through a 2 mm sieve and stones and organic debris removed. For (i), 5 g samples of soil were suspended each in 45 mL of sterile 0.05 % Tween-80 and shaken for 5 min at room temperature. The suspension was diluted in series to 10^{-4} in sterile 0.05 % Tween-80 and then 100 µL of the 10^{-3} and 10^{-4} suspension was plated in triplicate on selective media containing 1 % peptone, 1 % glucose, 1.25 % agar, and antibiotics chloramphenicol (50 mg/l) and cycloheximide (400 mg/l). The plates were incubated at 26 °C for 14 days. Colonies were sub-cultured on Sabouraud's dextrose agar (SDA) (Oxoid, ThermoFisher Scientific, United Kingdom) to obtain pure cultures. For (ii), insect-baiting method, we used wax moth larvae (*Galleria mellonella*) and mealworm larvae (*Tenebrio molitor*). 36 soil samples (200 g) were baited with either five, fifth instar wax moth larvae or five freshly shed mealworm larvae, using methods described by Inglis, Enkerli [48] and incubated at 26 °C and 70 % RH. After 13 to 17 days dead larvae were removed from soil, surface sterilised (70 % ethanol for 30 s, then rinsed three times in sterile distilled water), transferred to Petri dishes lined with sterile dampened filter paper, and incubated at 26 °C. Cadavers were monitored daily and fungal colonies were isolated from fungal hyphae emerging from the cadavers cultured on SDA agar as described previously. Cultures were initially identified using morphological keys [49, 50]. To compare isolates obtained from the Goulburn Valley region, we also tested two *Metarhizium* isolates obtained from soil in an almond block in the Mallee region of Victoria (collected as part of a separate study, August 2021), and one *Beauveria bassiana* and three *Metarhizium* isolates obtained from Queensland (Table 1).

Table 1. Details of entomopathogenic fungi evaluated against preimaginal life stages of Queensland fruit fly. Isolate codes de-identified.

Isolate Code	Identification	Isolate host	Origin
**BGV1**	*Beauveria bassiana*	Soil, selective agar	Goulburn Valley Region (Merrigum), VIC
**BQ1**	*B. bassiana*	*Musca domestica*	QLD
**MQ1**	*Metarhizium anisopliae*	Soil	South Johnstone, QLD
**MQ2**			
**MQ3**			Aratula, QLD
**MGV1**	*M. anisopliae*	Soil, *Galleria mellonella*	Goulburn Valley Region (Merrigum), VIC
**MGV2**			
**MGV3**			
**MGV4**	*M. anisopliae*	Soil, selective agar	
**MGV5**			
**MGV6**			
**MGV7**			
**MGV8**			
**MGV9**			
**MM1**	*M. anisopliae*	Soil, *Tenebrio molitor*	Mallee Region (Mildura), VIC
**MM2**			

Genomic DNA was extracted by the chemical lysis method using DNeasy® Plant Pro Kit (QIAGEN). The internal transcribed spacer (ITS) region was amplified using ITS5 (5'-GGAAGTAAAAGTCGTAACAAGG) and ITS4 (5'-TCCTCCGCTTATTGATATGC) primers [51]. Additionally, the elongation factor 1-alpha (EF1-α) region was also amplified using the primers 983F (5'-GCYCCYGGHCAYCGTGAYTTYAT) and 1567R (ACHGTRCCRATACCACCRAT) [52]. PCR master-mix was

prepared according to Qiagen AllTaq PCR core kit, with each 25 µL volume reactions with 3 µL of gDNA. Amplifications were carried with the following PCR cycling conditions after an initial denaturation step at 95 °C for 2 minutes, followed by 35 cycles of (i) denaturation at 95 °C for 5 seconds, (ii) annealing at 58 °C for 15 seconds, and (iii) extension at 72 °C for 10 seconds, and a final extension step at 72 °C for 2 minutes. The quality and size of a 5 µL PCR product were visualised on 1 % agarose gel PCR products were sequenced by Macrogen (South Korea). Phylogenetic analysis was carried out for the isolates using MEGA 11 [53] (data not shown). The use of the above primers identified all isolates as species within the *Metarhizium anisopliae* complex and hence described as *Metarhizium anisopliae sensu lato* (*M. anisopliae* s.l.).

Initial screening for pathogenicity of isolates against third-instar larvae

Two *B. bassiana* and 14 *M. anisopliae* s.l. isolates (Table 1) were screened against third-instar larvae of Queensland fruit fly. All isolates were grown on SDA for 21 days. Conidia were harvested from sporulating cultures by adding sterile 0.05 % Tween-80 and scraping spores with a sterile cell spreader. The resulting suspension was filtered through sterile Miracloth (22-25 µm; Millipore, Sigma-Adrich Pty. Ltd.) into a Falcon tube and vortexed for 1 min to homogenise. The concentration was calculated using a Neubauer Improved hemocytometer and adjusted to achieve a target application rate of 1×10^8 conidia g^{-1} vermiculite. The viability of conidia was evaluated for each isolate by plating a 10^{-6} dilution on SDA. Colony forming units (CFUs) were counted after seven days, and the viability was estimated [48]. The bioassay units consisted of transparent plastic vessels (946 mL, SteriCon™ 13, Austratec) containing 10 g of vermiculite (grade 1) each. The adjusted fungal suspensions were applied to each respective container by drenching with a pipette onto the vermiculite to achieve 33 % total water content. Inoculated vermiculite was subsequently mixed ~~thoroughly~~ in each container to ensure an even distribution of inoculum. The control was treated with sterile 0.05 % Tween-80 by the same procedure. To each container, a 60 mm Petri dish

containing Queensland fruit fly-infested carrot diet (26 g diet with approximately 39 eggs) was placed onto the vermiculite. Larvae were able to exit the diet to pupate in the treated substrate. The containers were covered with perforated lids (nine 1 mm holes) to allow gas exchange and incubated at 27 °C, with 60 % RH and 12:12 (L:D) photoperiod [36]. After 14 days of incubation, emerged flies were counted. Unemerged pupae were recovered, surface sterilised in 80 % ethanol for 30 s, followed by three rinses with sterile distilled water. Surface sterilised pupae were subsequently transferred onto 1% agar containing 0.5 g chloramphenicol / L for incubation to assess mycosis.

Assessing and comparing growth of fungal isolates

A series of experiments were conducted with three *Metarhizium* isolates (MGV1, MQ1, MM2) selected based on the initial pathogenicity assessment. The further aim was to compare these isolates for differences in hyphal growth, conidial germination, conidial yield, viability, and pathogenicity at different temperature regimes. To compare hyphal growth, conidial suspensions of 1 x 10^7 conidia mL^{-1} were prepared as previously described from 21-day old sporulating cultures of the three *Metarhizium* isolates. 100 µL of each suspension was spread onto SDA media and incubated at 27 °C for three days. A 5 mm mycelial mat was cut using a sterilised cork borer and transferred onto the centre of a fresh SDA plate for each isolate. The plates were previously marked with X and Y axes on the underside and then incubated at 21, 27, and 33 °C, respectively. The radial growth of each fungus was measured along the axes every second day for 21 days, with ten replicates per treatment for both the isolate treatment and temperature level. The maximum radial growth was reached when the isolate filled the Petri dish. Conidial yield was assessed for each isolate after 21 days by cutting three 100 mm circular disks with a sterile cork borer and suspending each in 10 mL sterile 0.05 % Tween-80. The suspension was mixed using a vortex for 1 min and the quantity of conidia estimated as conidia mL^{-1} using a haemocytometer. Each isolate and temperature

level were replicated in triplicate. The germination rate of each isolate was assessed by plating 20 µL of 1 x 10^{6} conidia mL^{-1} suspension on a thin film of SDA prepared on a glass slide. The glass slide was covered with a cover slip and incubated in a Petri dish on moist filter paper at the three temperature levels. Germination was quantified after 8, 10, 14, 18 and 24 hours by counting the first 100 conidia seen, this was replicated three times across different fields of view and for three slides per isolate. The conidia were considered germinated when the germ tube was twice the length of the conidium width [48, 54]. Additionally, spore viability was determined by plating 100 µL of the conidial suspension, diluted to 10^{-6} on SDA, incubating at 21, 27, and 33 °C, respectively, and counting CFUs after 5 days [26, 55]. To assess the virulence of the three isolates at different temperatures, groups of five third-instar larvae, were immersed in 1 mL of each suspension, prepared as previously described, at a concentration of 1 x 10^{7} conidia mL^{-1} and subsequently transferred onto 10 mL of sterile vermiculite (33 % water content). A control group was immersed in sterile 0.05 % Tween-80. After 2 days, the pupated larvae were transferred onto 1% agar (as described above) to assess mycosis. This bioassay was conducted twice with four replicates per isolate.

Pathogenicity against third-instar larvae and pupae of different ages

Bioassays were conducted to investigate the most susceptible juvenile stage of Queensland fruit fly, using the three candidate isolates MQ1, MGV1, and MM2, and a no-inoculum control, applied to sterile vermiculite as a pupation substrate according to the method previously described, except that substrate was prepared with a concentration of 9 x 10^{7} conidia g^{-1} vermiculite for the isolate treatments. Across six treatments, groups of 20 third-instar larvae, or 20 pupae with treatments at different ages (1, 3, 5, 7, and 9-day old) were transferred onto the treated vermiculite. Larvae were allowed to burrow naturally into the substrate, while pupae were carefully mixed into the vermiculite with a sterile hoop to ensure exposure to the respective substrate treatment. All bioassay containers were incubated as described previously and arranged in a complete randomised

nested split-plot design with three replicates per treatment, the whole bioassay was repeated three times (n=9). Units were observed daily and the number of flies that emerged recorded, pupae that failed to emerge were carefully recovered, surface sterilised and incubated as previously described to assess mycosis. Mortality by mycosis was verified after 3 to 5 days by observation of hyphal growth, and then after 7 to 10 days by observation of sporulating cadavers. In addition to estimating viability of the inoculum by quantifying the number of CFUs of the original suspension, the distribution of the inoculum in the vermiculite was assessed by suspending 1 g of inoculated vermiculite in 10 mL of 0.05 % Tween-80 from each experimental container. A 10^{-6} dilution was then prepared and plated onto SDA with three replicate plates per unit. After five days, the number of CFUs were counted to enable the calculation of viable spores per g^{-1} volume of vermiculite.

Survival of emerging adults

To investigate the susceptibility of emerging Queensland fruit fly adults to inoculated substrate, 12-day old pupae that were expected to emerge within 24 hours, were treated as previously described in the pupal treatments. Bioassay containers were monitored after 8, 12, 16 and 24 hours and any emerged flies were directly transferred into separate containers and supplied with water and sugar for nutrition. Flies were held at room temperature, and life span was recorded daily until 11 days after emergence. Cadavers were surface sterilised and incubated as previously described to access mycosis (Fig 1).

Fig 1. Photographs depicting mycosis of adult Queensland fruit fly, *Bactrocera tryoni,* inoculated each with three *Metarhizium anisopliae sensu lato* isolates, from left to right: isolates' MM2, MGV1, and MQ1.

Dose response in non-sterile orchard soil

This final bioassay investigated the concentration of inoculum required for fungal entomopathogens to infect third-instar larvae and achieve satisfactory rates of mortality, when exposed to non-sterile orchard soil. Soil was collected from the Agriculture Victoria SmartFarm at Tatura (Victoria), sieved through a 2 mm soil sieve to remove stones, organic debris, and insects, and then mixed with vermiculite in a 1:1 volume ratio for use as a pupation substrate in bioassays. The presence of resident fungal entomopathogens was first assessed with 15 soil samples according to the method described previously for isolating fungi from soil. *Metarhizium, Beauveria, Isaria, and Lecancillium* species were detected in the assessed soil samples, although the quantity was generally low (data not shown). The isolate MQ1 was prepared as an aqueous suspension of conidia in 0.01 % Tween-80 and adjusted to achieve the following concentrations: 1 x 10^6, 2.5 x 10^6, 5 x 10^6; 1 x 10^7, 2.5 x 10^7, and 5 x 10^7 conidia g^{-1} of soil, with 28 % soil moisture content. A no-inoculum control was prepared with 0.01 % Tween-80. Gravimetric soil moisture content was calculated by subtracting the weight of dry soil from the weight of wet soil, and then dividing by the weight of dry soil on the first day of the experiment and upon emergence of adult flies. The distribution of the inoculum in soil was also assessed as previously described by plating dilutions on selective agar media. The bioassay was incubated and arranged as described above with five replicates per treatment, and the entire bioassay was repeated three times (n=15).

Statistical Analysis

A probit model was used to analyse proportion mortality data of the initial pathogenicity screening and dose response assay. Specifically, a binominal generalised linear mixed model with a probit-link function was conducted using package lme4 [56] in R (R x64 4.2.2; RStudio). The fungal isolate factor was treated as a fixed effect in the model, and the bioassay experiment number was treated as a

random effect~~,~~ to account for any variation arising between experiments. Model assumptions were inspected by plotting residuals versus fitted values, and by testing for overdispersion based on Pearson's chi-squared residual test. Furthermore, a *post hoc* Tukey honesty significance differences (HSD) test was performed using a generalised linear hypotheses multiple comparison procedure using the package multcomp [57]. Mean percent mortalities were graphed with the package ggplot2 [58] to visualise and present the treatment effects. An ANOVA model or non-parametric Kruskal-Wallis (KW) test within the package agricolae [59] was used to analyse the percent mortality data of the life stage assay and the data of the temperature assay after testing the data for normal distribution and homogeneity of variances. Because older pupae ages (3-, 5-, 7-, and 9-day old) did not show mortality through fungal infection, the data was removed for statistical analysis. Percent mortality data, temperature data and trends of fungal germination were plotted over time with the package ggplot2. Viable rates of conidia were analysed with a regression model. A cox proportional-hazards model conducted with the packages survival and survminer [60] was used to analyse the survival of emerging adults after exposure to the fungal isolate treatment. A multiple log-rank test was used to compare survival curves by treatment group and plotted using the package survival.

Results

Initial pathogenicity screening of isolates against third-instar larvae

The two *B. bassiana* and 14 *M. anisopliae* s.l. isolates tested for pathogenicity against third-instar larvae showed significant differences in proportional mortality compared to the no-inoculum control ($F_{(16)} = 24.2; P < 0.001$), based on the probit model analysis (S1 Table). ~~Isolates MQ1, MQ2, and MQ3~~ were the most pathogenic to *B. tryoni* followed by MGV1, MM2, MM1, and ~~MGV3~~ (Fig 2). Overall, the *Beauveria* isolates were less pathogenic compared to the *Metarhizium* isolates; although only ~~isolates MQ1, MQ2, MQ3, and MGV1~~ were significantly different from the *Beauveria* isolates and no

inoculum-control. The viable conidial concentration applied differed between the isolates tested ($\chi^2_{(16)}$ = 239.3*; P < 0.00; KW*) and the bioassays conducted ($\chi^2_{(12)}$ = 72.7*; P < 0.01; KW*)~~,~~ However, the viable conidial concentrations applied were still within high rates of 9.29 x 10^6 - 1.55 x 10^8 conidia g^{-1} of vermiculite between the isolates tested.

Figure 2: Initial pathogenicity of 16 isolates tested (at 9 x 10^7 conidia g^{-1}) against ~~*B. tryoni* third-instar larvae~~. Bars represent the mean percentage mortality (± standard error). Different letters denote significant differences (p < 0.05) analysed on proportion data with generalised linear mixed model analysis with post-hoc Tukey HSD test.

Growth of selected fungal isolates

Radial growth rates were significantly different among temperature treatments (21 °C, 27 °C, and 33 °C) for the isolates tested ($\chi^2_{(8)}$ = 85.5*; P < 0.01; KW*). Isolate MQ1 reached maximum radial growth by 21 days at 21 °C and 27 °C and performed better at the higher temperature of 33 °C compared to MGV1 and MM2 (Table), whereas MGV1 only reached maximum growth at 27 °C. Overall, MM2 showed the lowest radial growth at all temperature levels tested. The conidia yield per 10 mm agar disc after 21 days showed significant differences between the isolates ($\chi^2_{(8)}$ = 24.9*; P = 0.002; KW*). The highest yield was recorded for isolate MQ1 at all three temperature levels, followed by MM2 and MGV1 (Table 2). Germination of conidia was significantly different among isolates when measured after 14 hours ($\chi^2_{(8)}$ = 24.6*; P = 0.002; KW*), with conidia of MQ1 germinating more rapidly with increasing temperatures (Fig. 3), whereas MM2 did not show a difference in germination rate between 27 °C and 33 °C (Table 2). MGV1 showed a significantly lower proportion of germinated conidia at 33 °C compared to 27 °C.

Fig. 3. Proportion of germinated conidia over 24 hours for MQ1, MM2, and MGV1 at different temperatures: (a) 21 °C, (b) 27 °C, and (c) 33 °C.

Table 2. Mean values for growth attributes and virulence of the three *M. anisopliae* s.l. isolates at different temperatures. Data represents means of replicates ± standard error. Treatments followed by different letters indicate significant differences (P < 0.05) based on non-parametric Kruskal-Wallis (KW)of percent data followed a Bonferroni post hoc analysis.

	**21 °C**				**27 °C**				**33 °C**			
	**MQ1**	**MM2**	**MGV1**	**0.05 % Tween-80**	**MQ1**	**MM2**	**MGV1**	**0.05% Tween-80**	**MQ1**	**MM2**	**MGV1**	**0.05 % Tween-80**
**Final colony diameter after 21 days [mm ± SE]**	89.20 ± 0.67 a	49.67 ± 0.32 d	71.19 ± 1.48 c	n/a	90.00 ± 0.00 a	69.11 ± 1.09 c	90.00 ± 0.00 a	n/a	75.78 ± 0.57 b	32.51 ± 0.52 e	35.57 ± 1.79 e	n/a
**Conidia yield after 21 days [x 10^7 conidia mL^{-1} ± SE]**	5.64 ± 1.33 a	1.27 ± 0.18 cd	0.41 ± 1.23 de	n/a	8.36 ± 0.81 a	4.19 ± 0.60 ab	2.16 ± 1.14 bc	n/a	2.38 ± 0.46 bc	0.00 ± 0.00 e	0.25 ± 0.66 de	n/a
**Proportion germinated conidia after 14 h ± SE**	0.40 ± 0.07 bc	0.11 ± 0.04 d	0.08 ± 0.01 d	n/a	0.67 ± 0.02 a	0.33 ± 0.01 bc	0.61 ± 0.03 ab	n/a	0.99 ± 0.01 a	0.33 ± 0.04 c	0.27 ± 0.01 cd	n/a
**Viability [%] after five days**	99	80	82	0	88	98	100	0	99	0	4	0
**Pupal mortality [% ± SE]**	75 ± 3.27 ab	90 ± 3.78 a	43 ± 9.60 bc	13 ± 13 de	90 ± 6.55 a	53 ± 8.40 bc	23 ± 5.90 cd	0 ± 0.00 e	100 ± 0.00 a	0 ± 0.00 e	25 ± 3.27 cd	0 ± 0.00 e

Effect of temperature on pathogenicity of selected isolates

Differences in virulence towards third-instar larvae were recorded among isolates and temperature levels ($\chi^2_{(11)} = 80.7$; $P < 0.001$; *KW*). At 21 °C MM2 showed a higher percentage of dead mycosed pupae, followed by MQ1. At 27 °C, MQ1 performed significantly better, followed by MM2. MGV1 showed the lowest percentage of mycosed pupae at 21 °C, with no significant difference observed at 27 °C and 33 °C, compared to MM2 and the untreated control. By contrast, MQ1 resulted in significantly higher insect mortality at 33 °C, while MM2 did not cause any fungal infection at 33 °C (Table). The percent viability calculated by CFUs ranged between 80 - 100 % depending on isolate and temperature level tested. Noteworthy, is that MM2 and MGV1 showed low viability of 0 % and 4 %, respectively, at 33 °C. Only MQ1 had viable conidia of 99 % at 33 °C after five days (Table). The plates of MM2 and MGV1 were incubated further at 27 °C for 5 days, and at this temperature, both isolates achieved viable rates of 82 % (MGV1) and 99 % (MM2).

Pathogenicity of selected isolates against different juvenile life stages

~~Pathogenicity~~ of ~~isolates MQ1, MM2, and MGV1~~ was investigated against third-instar larvae and pupae of different ages. Third-instar larvae were found to be the most susceptible group ($\chi^2_{(3)} = 31.6$; $P < 0.001$; *KW*) followed by one-day old pupae ($\chi^2_{(3)} = 9.7$; $P = 0.021$; *KW*). Isolate MQ1 achieved 98 % mortality when third-instar larvae were exposed to inoculated vermiculite; followed by MM2 and MGV1 with 78 % and 51 % mortality, respectively (Fig. 4). Percent conidial viability for the larvae treatments were 97 %, 94 %, and 95 % for MQ1, MM2, and MGV1. One-day old pupae were significantly less susceptible compared to the larval stage, with only 17 %, 13 %, and 10 % pupal mortality for isolates MM2, MQ1, and MGV1, respectively (Figure 4). Again, isolates showed high conidial viability of 97 % for MQ1, 94 % for MM2, and 95 % for MGV1.

Fig. 4. Pathogenicity of isolate MQ1, MM2 and MGV1 in vermiculite (9 x 10^7 conidia g^{-1}) against (a) third-instar larvae and (b) one-day old pupae of Queensland fruit fly. Controls were treated with sterile 0.05 % Tween-80. Bars represent the mean percent mortality (± standard error). Different letters denote significant differences between isolate treatments and the no-inoculum control.

Survival of treated emerging adults

Emerging Queensland fruit fly adults were susceptible to all inoculum treatments and significant differences were found between isolates (Fig. 5). The Cox proportional-hazards model used to investigate the survival probability of adult flies over 10 days indicated that ~~isolate MQ1 and MM2~~ were significantly more virulent in the first 10 days after emergence~~,~~ compared to MGV1 and the untreated control (*likelihood ratio test = 132.8; df = 3;* $p < 0.00$). The percent mortalities after 9 days were 16.9 %, 56.3 %, 66.1%, 75.7 % for the untreated control, MGV1, MM2, and MQ1, respectively. The conidial viability of the inoculum was 97 % for MQ1, 89 % for MM2, and 83 % for MGV1.

Fig. 5: Survival curves of adult Queensland fruit fly emerged from fungal isolate treated vermiculite (9 x 10^7 conidia g^{-1}) showing the time-mortality response. Survival curves are analysed using Cox proportional-hazards model followed by different letters denote significant differences (*$p < 0.05$*) with a multiple comparison log-rank test.

Dose response in non-sterile orchard soil

When MQ1 was applied to non-sterile soil the linear regression model indicated a significant positive relationship between proportion of mycosed pupae and increasing fungal concentration applied mortality (adjusted $R^2 = 0.85$; $F(1, 103) = 581.1$; $P < 0.01$). The slope of the fit between the viable rate

applied ~~on~~ proportion mortality rate was 1.223^{-08} with an intercept of 9.127^{-02}, indicating that a tenfold increase in concentration had ~11 % increase in mortality. Concentrations higher than 2.5 x 10^6 conidia g^{-1} soil showed significant differences in pupal mortality compared to untreated control ($F_{(6)} = 37.4; p < 0.001$). The highest percent pupal mortalities of 66 % mortality were achieved with the highest concentration of 5 x 10^7 conidia g^{-1} soil (Fig. 6). Between the experiments conducted the viability of the isolate MQ1 ranged between 90 - 98%. The soil moisture content was consistent throughout the experiment with minimal moisture loss of 4.1 %. Moreover, the fungal concentration $gram^{-1}$ of dry soil observed was within the range of the estimated dosage applied.

Fig. 6. (a) Mean percentage pupal mortality of the isolate MQ1 towards third-instar larvae of Queensland fruit fly in non-sterile orchard soil. Data represents means of replicates ± standard error. Treatments followed by different letters indicate significant difference (*p < 0.05*) through generalised linear mixed model analysis of proportional data followed a Tukey HSD test. (b) Linear regression for proportion juvenile mortality with 95% confidence interval indicated in grey

$$\left(\mathbf{9.127^{-02}} + \left(\mathbf{1.223^{-08}} \times \boldsymbol{viable\ rate\ applied}\right) + \mathbf{0.084}\right).$$

Discussion

Isolates of *B. bassiana* and *M. anisopliae* s.l. species were evaluated for pathogenicity in soil-dwelling juvenile stages of Queensland fruit fly, to explore the potential use of these fungi as biopesticide agents. In initial screening trials, juvenile life stages were found to be highly susceptible to infection by the two fungal genera tested. *M. anisopliae* s.l. isolates showed higher pathogenicity to vulnerable life stages of Queensland fruit fly compared to the *Beauveria* isolates, with significant differences observed among isolates. Mortality rates in our study were comparable to those in studies screening entomopathogenic fungi against other tephritid fruit fly species [29-31, 34, 35, 40,

61]. For example, pathogenicity studies using *M. anisopliae* against larvae of the carambola fruit fly (*B. carambolae*) demonstrated mortality of 70 % in pre-pupating larvae in sterile soil [38]. A study screening 20 different isolates of *M. anisopliae* against third-instar larvae of the Mexican fruit fly (*Anastrepha ludens*) demonstrated mortalities ranging from 37.5 - 98.75 % [26].

Our results showed mortality varying from 26 – 93 % depending on the tested *Metarhizium* isolate. Both of the other studies mentioned above used the same high fungal concentration, while the application method differed between the studies. Our study incorporated the fungal suspension in the substrate, whereas these other two studies sprayed the suspension into the soil or applied the fungal entomopathogen through immersion of the larvae in a fungal suspension. Our method might therefore better represent the pest ecology and intended application environment [16].

Pathogenicity can vary between fungal species, strains, and even isolates [62]. For example, screening conducted against the peach fruit fly (*B. zonata*) and the Oriental fruit fly (*B. dorsalis*) demonstrated that *B. bassiana* was more effective compared to *Metarhizium* isolates tested [35, 40], which differs from our findings on Queensland fruit fly. Fungal species and strain selection ~~is~~ therefore important to consider in terms future development of fungal entomopathogens to control a particular fruit fly pest [16].

Our study is the first to test the susceptibility of soil-dwelling life stages of Queensland fruit fly (third-instar larvae and pupae) following exposure to *M. anisopliae* s.l. isolates. Our initial pathogenicity study revealed the susceptibility of the soil-dwelling life stages of Queensland fruit fly to the ~~tested isolates~~. However, the study provided no indication of when infection in soil occurred, the duration of the incubation period, and which life stage (larvae, pupae) was susceptible to infection. Thus, we also tested the susceptibility of different age groups in order to better understand when infection can occur. Pre-pupating third-instar larvae were the most susceptible soil-dwelling life stage to entomopathogenic fungi, which is consistent with what has been observed

in other tephritid fruit fly species [30, 35, 40, 62, 63]. Our findings showed that one-day old pupae were comparatively less susceptible than larvae but were nonetheless vulnerable. These findings differ from a study on the peach fruit fly (*B. zonata*), which did not observe significant mortality when fresh pupae were inoculated by spray or inoculated in sterile soil with *B. bassiana*, *M. anisopliae,* or *Isaria fumosorosea* in different concentrations (1 x, 2 x, and 3 x 10^8) [40]; however the age of the pupae was not precisely determined in that instance.

Older pupal ages did not show any sign of fungal infection in our study. The greater melanisation and sclerotisation of the mature pupal cuticle may prevent or limit the ability of fungal entomopathogens to infect older pupae [64]. This indicates a limited period for the pre-pupal stage to encounter sufficient fungal spores to succumb to infection. By contrast, significant mortality has been demonstrated when 4 to 5-day old pupae of the peach fruit fly (*B. zonata*) and the Oriental fruit fly (*B. dorsalis*) are exposed to *B. bassiana* isolates or *M. anisopliae* isolates in sterile soil treated with concentrations of 1 x 10^7 and 1 x 10^8 conidia mL^{-1} [35]. *Beauveria* isolates caused higher mortalities in both fly species compared to the *Metarhizium* isolates. However, the study used cumulative mortality of pupae and ~~adult's~~ post-emergence when pupae were treated. ~~Results~~ may therefore have been confounded by the emerging adults becoming infected during pupal eclosion. We also observed mortality of adult flies emerging from treated pupae, and subsequently investigated infection of new adults by exposing 12-day old Queensland fruit fly pupae. Adults emerging from this pupal treatment had a significant decrease in survival within the first 10 days. Mortalities ranged between 56 – 76 %, depending on the isolate tested. By comparison, within five days post-emergence, 100 % adult mortality was reported when the late instar larvae of the carambola fruit fly (*B. carambolae*) were exposed to *M. anisopliae* [38]. The longer time of exposure to the inoculum from late instar ~~larvae~~ stage until post-emergence is likely to cause higher mortalities ~~observed~~ compared to what we observed in this present study. Soil-dwelling life stages of

Queensland fruit fly can therefore be targeted when entering the soil as larvae and exiting the soil during adult emergence.

As tephritid fruit flies emerge as sexually immature adults, this provides an opportunity to kill adults before they reach maturation. *Bactrocera* species reach sexual maturity around 10 days after emergence under laboratory conditions [4]. For Queensland fruit fly*,* maturation periods from 5 to 31 days have been reported, depending on rearing history and conditions [4], although maturation periods under natural conditions may vary. We provided evidence that the tested *Metarhizium* isolates resulted in a significant reduction in adult survival within the first 10 days following exposure to the inoculum, if no infection occurs prior to pupation. Adults that survive treatment upon maturation may show a reduction in fecundity, as observed in females of the Mexican fruit fly (*A. ludens*) were treated with *B. bassiana*, and females of the Mediterranean fruit fly (*C. capitata*) when exposed to *M. anisopliae* [28, 33].

Environmental temperature can strongly influence efficacy, longevity, growth, spore production, germination, pathogenicity, and virulence of fungal entomopathogens towards different hosts and has proven to be a critical factor for their success in the field [43, 44, 65-67]. Our findings demonstrated that the ~~isolate~~ MQ1 had the highest potential at the temperature levels tested~~,~~ by achieving the highest radial growth rate, highest germination rate after 14 hours, with highest conidial yield, in addition to high conidial viability, when measured by the number of CFUs. Generally, the optimum temperature for fungal entomopathogen growth and germination ranges between 25 – 30 °C [68]. Studies often investigate the viability of a specific isolate by simply counting germinated spores [68-71]. Besides the germination rate, however, we also investigated the viability of isolates by quantifying the number of CFUs that were able to grow at the temperature tested. At 33 °C only MQ1 showed viable colonies whereas MM2 and MGV1 did not, even when the isolates showed germination rates over 50 % after 24 h. Interestingly, when the cultures were moved to 27 °C, these isolates resumed growth and showed high viability by CFU counts. These observations were

comparable with a study that tested upper temperature limits of *Metarhizium* species [72]. After exposure to high temperatures for 10 days, some isolates were able to resume growth when transferred to lower temperatures. This indicates that testing germination rate might not be sufficient to report on an ~~isolates~~ viability.

It is widely recognised that high temperatures have a negative effect on conidial germination and viability [71, 73, 74] but the decrease in fungal efficiency at high temperatures is also possibly associated with ~~increase~~ stress responses of fungal isolates [75, 76] or due to the thermal response of the insect host [77, 78], rather than conidia germination and fungal growth rate directly. The current study assessed the virulence towards third-instar larvae of Queensland fruit fly. Again, ~~isolate~~ MQ1 showed the highest potential across the three temperatures tested. However, the ~~isolate~~ MM2 showed slightly higher mortalities in third-instar larvae at 21 °C~~,~~ compared to MQ1. Though this difference was not significant it may indicate that MM2 might perform better at cooler temperatures compared to the other isolates, though we have not tested below the optimal temperature range in this study. Different fungal entomopathogen species and isolates can show differences in their optimal temperature range [70], [79] and thus might be more suitable for application in different geographical regions and during the seasons to maximise their efficiency in the field [43, 44]. Further work might explore regional and seasonal soil temperatures relevant in Australia, which covers a large range of climate zones, from tropical to temperate and arid regions.

Besides temperature, soil microbiota, moisture content, structure, and chemistry also influence the availability and activity of fungal spore activity, which causes variability in host specific fungal infection in soil compared to vermiculite or other sterile substrate [44, 80]. This host specificity can additionally be dose-dependent [81] and ultimately alter the number of spores needed; hence, the dosage needed to achieve notable effect on a pest population. ~~Isolate~~ MQ1 was tested at different dosages in non-sterile orchard soil. Our results revealed that MQ1 achieved up to 66 % pupal mortality when third-instar larvae were exposed to high concentrations of 1 x 10^7 conidia g^{-1}. Similar

mortality rates of 40 - 83.3 % were reported when *M. anisopliae* was incorporated in non-sterile soil to control Mediterranean fruit fly (*C. capitata*) [80]. By contrast, *M. anisopliae* and *M. robertsii* sprayed on non-sterile soil (1 x 10^8 conidia mL^{-1}) was only able to achieve 28 % and 44 % mortality, respectively, against larvae of the carambola fruit fly (*B. carambolae*) [38]. In these studies, soil water content, which is known to influence an isolates pathogenicity [30], was maintained by periodically moistening the soil. We controlled the water content in soil during the experiment which was relatively constant and optimum at 26 % and therefore likely had little impact on our mortality results. It is well known that fungal entomopathogens generally achieve lower mortalities in non-sterile soil compared to sterile soil; primarily this may be attributed due to the influence of the resident soil microbiota [44]. The non-sterile orchard soil we used contained low levels of (different) fungal entomopathogens and interactions within the soil microbiota may have occurred, which could cause some reduction in the efficiency [82-85]. However, the mycosis observed on cadavers appeared to be caused by the inoculum treatment, based on morphological characteristics.

We provide evidence that *M. anisopliae* s.l. isolates show considerable potential as a soil-applied biocontrol agent against Queensland fruit fly. The use of fungal entomopathogens could be incorporated into Integrated Pest Management (IPM) and Area Wide Management (AWM) strategies to enable more holistic control of this pest, decreasing populations of Queensland fruit fly that evade current control. Soil applied formulations could have particular use for targeting larvae emerging from fruit left on the ground at the end of the season; these populations being the source of spring populations. Future work to progress the development of fungal entomopathogens as biopesticides could look at formulation, in-field persistence, and pathogenicity to non-target insects.

Acknowledgements

The authors would like to thank William Boston for his support during the isolation process of fungal entomopathogens.

References

1. Dominiak BC, Daniels D. Review of the past and present distribution of Mediterranean fruit fly (Ceratitis capitata Wiedemann) and Queensland fruit fly (Bactrocera tryoni Froggatt) in Australia. Australian Journal of Entomology. 2012;51(2):104-15. doi:10.1111/j.1440-6055.2011.00842.x
2. Sutherst RW, Collyer BS, Yonow T. The vulnerability of Australian horticulture to the Queensland fruit fly, Bactrocera (Dacus) tryoni, under climate change. Australian Journal of Agricultural Research. 2000;51(4):467-80. doi:10.1071/AR98203
3. Clarke AR, Powell KS, Weldon CW, Taylor PW. The ecology of Bactrocera tryoni (Diptera: Tephritidae): what do we know to assist pest management? Annals of Applied Biology. 2011;158(1):26-54.
4. Clarke AR. Biology and Management of Bactrocera and Related Fruit Flies: CABI; 2019.
5. Sultana S, Baumgartner JB, Dominiak BC, Royer JE, Beaumont LJ. Impacts of climate change on high priority fruit fly species in Australia. PloS one. 2020;15(2):e0213820. doi:10.1371/journal.pone.0213820
6. APVMA. Dimethoate Chemical Review 2016 [Available from: https://apvma.gov.au/dimethoate.
7. APVMA. Fenthion Chemical Review 2014 [Available from: https://apvma.gov.au/node/12541.
8. Henneken J, Farnier K, Cunningham JP. A Synthetic Blend of Fruit and Live Yeast Odours Shows Promise for Trapping Mated Female Queensland Fruit Fly, Bactrocera tryoni, in the Field. Journal of Chemical Ecology. 2022:1-10. doi:10.1007/s10886-022-01387-x
9. Meats A, Duthie R, Clift A, Dominiak B. Trials on variants of the Sterile Insect Technique (SIT) for suppression of populations of the Queensland fruit fly in small towns neighbouring a quarantine (exclusion) zone. Australian Journal of Experimental Agriculture. 2003;43(4):389-95. doi:10.1071/EA02064
10. Dominiak BC. Components of a systems approach for the management of Queensland fruit fly Bactrocera tryoni (Froggatt) in a post dimethoate fenthion era. Crop Protection. 2019;116:56-67. doi:10.1016/j.cropro.2018.10.002
11. Ekesi S, Maniania N, Mohamed S. Efficacy of soil application of Metarhizium anisopliae and the use of GF-120 spinosad bait spray for suppression of Bactrocera invadens (Diptera: Tephritidae) in mango orchards. Biocontrol Science and Technology. 2011;21(3):299-316. doi:10.1080/09583157.2010.545871

12. Aryal S, Nielsen UN, Sumaya NH, Wilson C, Riegler M. Virulence, penetration rate and reproductive potential of entomopathogenic nematodes from eastern Australia in Queensland fruit fly, Bactrocera tryoni. Biological Control. 2022;169:104871. doi:10.1016/j.biocontrol.2022.104871
13. Clarke AR, Harris C, Kay BJ, Mainali BP, McLay LK, Strutt F, et al. Opiine parasitoids (Hymenoptera: Braconidae) and biological control of fruit flies (Diptera: Tephritidae) in Australia: Past, present and future. Annals of Applied Biology. 2022;180(1):44-72. doi:10.1111/aab.12724
14. Harris C, Bromley E, Clarke LK, Kay BJ, Schwenke AC, Clarke AR. Conservation biological control of the fruit fly parasitoid Fopiusa arisanus (Hymenoptera: Braconidae). Austral Entomology. 2022;61(3):340-9. doi:10.1111/aen.12614
15. De Faria MR, Wraight SP. Mycoinsecticides and mycoacaricides: a comprehensive list with worldwide coverage and international classification of formulation types. Biological control. 2007;43(3):237-56. doi:10.1016/j.biocontrol.2007.08.001
16. Glare T, McKinnon A, Gwynn R, Brownbridge M. Advances in microbial control in integrated pest management: entomopathogenic fungi. Integrated management of insect pests: Current and future developments: Burleigh Dodds Science Publishing; 2019. p. 549-92.
17. Dias NP, Montoya P, Nava DE. A 30-year systematic review reveals success in tephritid fruit fly biological control research. Entomologia Experimentalis et Applicata. 2022;170(5):370-84. doi:10.1111/eea.13157
18. Zimmermann G. Review on safety of the entomopathogenic fungus Metarhizium anisopliae. Biocontrol Science and Technology. 2007;17(9):879-920.
19. Zimmermann G. Review on safety of the entomopathogenic fungi Beauveria bassiana and Beauveria brongniartii. Biocontrol Science and Technology. 2007;17(6):553-96.
20. Mascarin GM, Jaronski ST. The production and uses of Beauveria bassiana as a microbial insecticide. World Journal of Microbiology and Biotechnology. 2016;32(177):1-26. doi:10.1007/s11274-016-2131-3
21. Inglis G, Goettel M, Butt TM, Strasser H. Use of Hyphomycetous Fungi for Managing Insect Pests. Fungi as biocontrol agents: progress, problems and potential: CABI Publisher; 2001.
22. Yousef M, Alba-Ramírez C, Garrido Jurado I, Mateu J, Raya Diaz S, Valverde-Garcia P, et al. Metarhizium brunneum (Ascomycota; Hypocreales) treatments targeting olive fly in the soil for sustainable crop production. Frontiers in Plant Science. 2018;9:1. doi:10.3389/fpls.2018.00001
23. Soliman N, Al-amin SM, Mesbah AE, Ibrahim AM, Mahmoud AM. Pathogenicity of three entomopathogenic fungi against the Mediterranean fruit fly, Ceratitis capitata (Wiedemann)(Diptera: Tephritidae). Egyptian Journal of Biological Pest Control. 2020;30(49):1-8. doi:10.1186/s41938-020-00235-y
24. Carswell I, Spooner-Hart R, Milner RJ. Laboratory susceptibility of Musca domestica L.(Diptera: Muscidae) and Bactrocera tryoni (Frogatt)(Diptera: Tephritidae) to an isolate of Metarhizium anisopliae (Metsch.) Sorokin. Australian journal of Entomology. 1998;37(3):281-4. doi:10.1111/j.1440-6055.1998.tb01584.x
25. Vega FE, Posada F, Aime MC, Pava-Ripoll M, Infante F, Rehner SA. Entomopathogenic fungal endophytes. Biological control. 2008;46(1):72-82. doi:10.1016/j.biocontrol.2008.01.008

26. Lezama-Gutiérrez R, la Luz AT-d, Molina-Ochoa J, Rebolledo-Dominguez O, Pescador AR, López-Edwards M, et al. Virulence of Metarhizium anisopliae (Deuteromycotina: Hyphomycetes) on Anastrepha ludens (Diptera: Tephritidae): laboratory and field trials. Journal of Economic Entomology. 2000;93(4):1080-4. doi:10.1603/0022-0493-93.4.1080
27. Wilson WM, Ibarra JE, Oropeza A, Hernández MA, Toledo-Hernández RA, Toledo J. Infection of Anastrepha ludens (Diptera: Tephritidae) adults during emergence from soil treated with Beauveria bassiana under various texture, humidity, and temperature conditions. Florida Entomologist. 2017;100(3):503-8. doi:10.1653/024.100.0302
28. Toledo J, Campos SE, Flores S, Liedo P, Barrera JF, Villaseñor A, et al. Horizontal transmission of Beauveria bassiana in Anastrepha ludens (Diptera: Tephritidae) under laboratory and field cage conditions. Journal of economic entomology. 2007;100(2):291-7.
29. De la Rosa W, López F, Liedo P. Beauveria bassiana as a pathogen of the Mexican fruit fly (Diptera: Tephritidae) under laboratory conditions. Journal of Economic Entomology. 2002;95(1):36-43. doi:10.1603/0022-0493-95.1.36
30. Quesada-Moraga E, Ruiz-García A, Santiago-Alvarez C. Laboratory evaluation of entomopathogenic fungi Beauveria bassiana and Metarhizium anisopliae against puparia and adults of Ceratitis capitata (Diptera: Tephritidae). Journal of Economic Entomology. 2006;99(6):1955-66. doi:10.1093/jee/99.6.1955
31. Ekesi S, Maniania N, Lux S. Mortality in three African tephritid fruit fly puparia and adults caused by the entomopathogenic fungi, Metarhizium anisopliae and Beauveria bassiana. Biocontrol Science and Technology. 2002;12(1):7-17. doi:10.1080/09583150120093077
32. Dimbi S, Maniania NK, Lux SA, Ekesi S, Mueke JK. Pathogenicity of Metarhizium anisopliae (Metsch.) Sorokin and Beauveria bassiana (Balsamo) Vuillemin, to three adult fruit fly species: Ceratitis capitata (Weidemann), C. rosa var. fasciventris Karsch and C. cosyra (Walker)(Diptera: Tephritidae). Mycopathologia. 2003;156(4):375-82.
33. Castillo M-A, Moya P, Hernández E, Primo-Yufera E. Susceptibility of Ceratitis capitata Wiedemann (Diptera: Tephritidae) to entomopathogenic fungi and their extracts. Biological control. 2000;19(3):274-82. doi:10.1006/bcon.2000.0867
34. Yousef M, Lozano-Tovar MD, Garrido-Jurado I, Quesada-Moraga E. Biocontrol of Bactrocera oleae (Diptera: Tephritidae) With Metarhizium brunneum and Its Extracts. Journal of Economic Entomology. 2013;106(3):1118-25. doi:10.1603/ec12489
35. Usman M, Wakil W, Piñero JC, Wu S, Toews MD, Shapiro-Ilan DI. Evaluation of Locally Isolated Entomopathogenic Fungi against Multiple Life Stages of Bactrocera zonata and Bactrocera dorsalis (Diptera: Tephritidae): Laboratory and Field Study. Microorganisms. 2021;9(8):1791. doi:10.3390/microorganisms9081791
36. Shahzad M, Qayyum M, Ishtiaq M, Ikram R, Saleem M. Entomopathogenicity of Beauveria Bassiana against two Bactrocera Species (Tephritidae: Diptera) under Laboratory Condition. Agricultural Sciences Journal. 2019;1(1):19-24.
37. Silva TdL, Lima AL, de Sousa MdSM, de Jesus-Barros CR, Bariani A, Pereira JF, et al. Potential of Amazonian isolates of Metarhizium to control immatures of Bactrocera carambolae (Diptera: Tephritidae). Florida Entomologist. 2016;99(4):788-9. doi:10.1653/024.099.0436
38. Brito BD, Lima AL, Cruz KR, Bariani A, Jesus-Barros CR, Pereira JF, et al. Amazonian isolates of Metarhizium are effective for killing Bactrocera carambolae (DIPTERA:

TEPHRITIDAE). Acta Biológica Colombiana. 2019;24(1):118-24. doi:10.15446/abc.v24n1.70275

39. Rashad MM, El-Heneidy A, Djelouah K, Hassan N, Shaira SA. On the pathogenicity of entomopathogens to the peach fruit fly, Bacterocera zonata (Saunders)(Diptera: Tephritidae). Egyptian Journal of Biological Pest Control. 2015;25(3).

40. Gul HT, Freed S, Akmal M, Malik MN. Vulnerability of different life stages of Bactrocera zonata (Tephritidae: Diptera) against entomogenous fungi. Pakistan Journal of Zoology. 2015;47(2).

41. Butt T, Goettel M. Bioassays of entomogenous fungi. In: Navon A, Ascher KRS, editors. Bioassays of entomopathogenic microbes and nematodes: CABI; 2000. p. 141-95.

42. Meyling NV, Eilenberg J. Ecology of the entomopathogenic fungi Beauveria bassiana and Metarhizium anisopliae in temperate agroecosystems: potential for conservation biological control. Biological control. 2007;43(2):145-55. doi:10.1016/j.biocontrol.2007.07.007

43. Keyser CA. Development of a Laboratory Based System for Selecting Insect Pathogenic Fungi with Greatest Potential for Success in the Field. Utah State University, Utha2010.

44. Jaronski ST. Ecological factors in the inundative use of fungal entomopathogens. BioControl. 2010;55(1):159-85. doi:10.1007/s10526-009-9248-3

45. Cory JS, Ericsson JD. Fungal entomopathogens in a tritrophic context. The ecology of fungal entomopathogens: Springer; 2009. p. 75-88.

46. Beris EI, Papachristos DP, Fytrou A, Antonatos SA, Kontodimas DC. Pathogenicity of three entomopathogenic fungi on pupae and adults of the Mediterranean fruit fly, Ceratitis capitata (Diptera: Tephritidae). Journal of Pest Science. 2013;86(2):275-84. doi:10.1007/s10340-012-0468-4

47. Zimmermann G. The 'Galleria bait method'for detection of entomopathogenic fungi in soil. Journal of applied Entomology. 1986;102(1-5):213-5.

48. Inglis GD, Enkerli J, Goettel MS. Laboratory techniques used for entomopathogenic fungi: Hypocreales. Manual of techniques in invertebrate pathology. 2012;2:18-53.

49. Humber RA. Identification of entomopathogenic fungi. Manual of techniques in invertebrate pathology. 2012:151-87.

50. Barnett HL, Hunter BB. Illustrated genera of imperfect fungi. Illustrated genera of imperfect fungi. 1972(3rd ed).

51. White TJ, Bruns T, Lee S, Taylor J. Amplification and direct sequencing of fungal ribosomal RNA genes for phylogenetics. PCR protocols: a guide to methods and applications. 1990;18(1):315-22.

52. Rehner SA, Buckley E. A Beauveria phylogeny inferred from nuclear ITS and EF1-α sequences: evidence for cryptic diversification and links to Cordyceps teleomorphs. Mycologia. 2005;97(1):84-98.

53. Tamura K, Stecher G, Kumar S. MEGA11: molecular evolutionary genetics analysis version 11. Molecular biology and evolution. 2021;38(7):3022-7. doi:10.1093/molbev/msab120

54. Jaber LR, Araj S-E. Interactions among endophytic fungal entomopathogens (Ascomycota: Hypocreales), the green peach aphid Myzus persicae Sulzer (Homoptera: Aphididae), and the aphid endoparasitoid Aphidius colemani Viereck (Hymenoptera: Braconidae). Biological Control. 2018;116:53-61. doi:10.1016/j.biocontrol.2017.04.005

55. Lacey LA, Martins A, Ribeiro C. The pathogenicity of Metarhizium anisopliae and Beauveria bassiana for adults of the Japanese beetle, Popillia japonica (Coleoptera: Scarabaeidae). European Journal of Entomology. 1994;91(3):313-9.
56. Bates D, Mächler M, Bolker B, Walker S. Fitting linear mixed-effects models using lme4. arXiv preprint arXiv:14065823. 2014.
57. Hothorn T, Bretz F, Westfall P, Heiberger RM, Schuetzenmeister A, Scheibe S, et al. Multcomp: Simultaneous inference in general parametric models. Project for Statistical Computing. 2023.
58. Wickham H, Chang W, Wickham MH. Ggplot2: Create elegant data visualisations using the grammar of graphics. 2014;2(1):1-189.
59. Mendiburu Fd. Agricolae: statistical procedures for agricultural research. (No Title). 2023.
60. Therneau T. A package for survival analysis in R. R package version. 2015;2(7).
61. Sookar P, Bhagwant S, Allymamod M. Mortality in Tephritid Fruit Fry Puparia and Adults Caused by Metarhizium anisopliae, Paecilomyces fumosoroseus and Beauveria bassiana. University of Mauritius Research Journal. 2010;16(1):281-98.
62. Cossentine J, Jaronski S, Thistlewood H, Yee W. Impact of Metarhizium brunneum (Hypocreales: Clavicipitaceae) on pre-imaginal Rhagoletis indifferens (Diptera: Tephritidae) within and on the surface of orchard soil. Biocontrol Science and Technology. 2011;21(12):1501-5. doi:10.1080/09583157.2011.625397
63. Carneiro RMDG, Salles LAB. Pathogenicity of Paecilomyces fumosoroseus isolate CG 260 on larvae and pupae of Anastrepha fraterculus Wied. Anais da Sociedade Entomológica do Brasil. 1994;23(2):341-3.
64. Ortiz-Urquiza A, Keyhani NO. Action on the surface: entomopathogenic fungi versus the insect cuticle. Insects. 2013;4(3):357-74. doi:10.3390/insects4030357
65. Mishra S, Kumar P, Malik A. Effect of temperature and humidity on pathogenicity of native Beauveria bassiana isolate against Musca domestica L. Journal of parasitic diseases. 2015;39(4):697-704. doi:10.1007/s12639-013-0408-0
66. Bugeme DM, Knapp M, Boga HI, Wanjoya AK, Maniania NK. Influence of temperature on virulence of fungal isolates of Metarhizium anisopliae and Beauveria bassiana to the two-spotted spider mite Tetranychus urticae. Mycopathologia. 2009;167(4):221-7. doi:10.1007/s11046-008-9164-6
67. Athanassiou CG, Kavallieratos NG, Rumbos CI, Kontodimas DC. Influence of temperature and relative humidity on the insecticidal efficacy of Metarhizium anisopliae against larvae of Ephestia kuehniella (Lepidoptera: Pyralidae) on wheat. Journal of insect science. 2017;17(1). doi:10.1093/jisesa/iew107
68. Ekesi S, Maniania N, Ampong-Nyarko K. Effect of temperature on germination, radial growth and virulence of Metarhizium anisopliae and Beauveria bassiana on Megalurothrips sjostedti. Biocontrol Science and Technology. 1999;9(2):177-85. doi:10.1080/09583159929767
69. Ekesi S, Maniania N, Lux S. Effect of soil temperature and moisture on survival and infectivity of Metarhizium anisopliae to four tephritid fruit fly puparia. Journal of Invertebrate Pathology. 2003;83(2):157-67. doi:10.1016/S0022-2011(03)00069-7
70. Dimbi S, Maniania NK, Lux SA, Mueke JM. Effect of constant temperatures on germination, radial growth and virulence of Metarhizium anisopliae to three species of African tephritid fruit flies. BioControl. 2004;49(1):83-94.

71. Liu H, Skinner M, Brownbridge M, Parker BL. Characterization of Beauveria bassiana and Metarhizium anisopliae isolates for management of tarnished plant bug, Lygus lineolaris (Hemiptera: Miridae). Journal of Invertebrate Pathology. 2003;82(3):139-47. doi:10.1016/S0022-2011(03)00018-1

72. Rangel DE, Fernandes ÉK, Dettenmaier SJ, Roberts DW. Thermotolerance of germlings and mycelium of the insect-pathogenic fungus Metarhizium spp. and mycelial recovery after heat stress. Journal of Basic Microbiology. 2010;50(4):344-50. doi:10.1002/jobm.200900430

73. McClatchie G, Moore D, Bateman R, Prior C. Effects of temperature on the viability of the conidia of Metarhizium flavoviride in oil formulations. Mycological Research. 1994;98(7):749-56. doi:10.1016/S0953-7562(09)81049-6

74. De Oliveira DGP, Lopes RB, Rezende JM, Delalibera Jr I. Increased tolerance of Beauveria bassiana and Metarhizium anisopliae conidia to high temperature provided by oil-based formulations. Journal of invertebrate pathology. 2018;151:151-7. doi:10.1016/j.jip.2017.11.012

75. Lovett B, Leger RJS. Stress is the rule rather than the exception for Metarhizium. Current Genetics. 2015;61(3):253-61. doi:10.1007/s00294-014-0447-9

76. Keyser CA, Fernandes ÉK, Rangel DE, Roberts DW. Heat-induced post-stress growth delay: a biological trait of many Metarhizium isolates reducing biocontrol efficacy? Journal of invertebrate pathology. 2014;120:67-73. doi:10.1016/j.jip.2014.05.008

77. Xie Q, Hou B, Zhang R. Thermal responses of oriental fruit fly (Diptera: Tephritidae) late third instars: mortality, puparial morphology, and adult emergence. Journal of Economic Entomology. 2008;101(3):736-41. doi:10.1093/jee/101.3.736

78. Yu C, Zhao R, Zhou W, Pan Y, Tian H, Yin Z, et al. Fruit fly in a challenging environment: Impact of short-term temperature stress on the survival, development, reproduction, and trehalose metabolism of Bactrocera dorsalis (Diptera: Tephritidae). Insects. 2022;13(8):753. doi:10.3390/insects13080753

79. Rivas F, Nuñez P, Jackson T, Altier N. Effect of temperature and water activity on mycelia radial growth, conidial production and germination of Lecanicillium spp. isolates and their virulence against Trialeurodes vaporariorum on tomato plants. BioControl. 2014;59(1):99-109. doi:10.1007/s10526-013-9542-y

80. Garrido-Jurado I, Torrent J, Barrón V, Corpas A, Quesada-Moraga E. Soil properties affect the availability, movement, and virulence of entomopathogenic fungi conidia against puparia of Ceratitis capitata (Diptera: Tephritidae). Biological Control. 2011;58(3):277-85. doi:10.1016/j.biocontrol.2011.05.017

81. Milner RJ. Current status of Metarhizium as a mycoinsecticide in Australia. Biocontrol News and Information. 2000;21(2):47N-50N.

82. Jaronski ST. Soil ecology of the entomopathogenic ascomycetes: a critical examination of what we (think) we know. Use of entomopathogenic fungi in biological pest management. 2007:91-143.

83. Sharma S, Gupta R. Effect of certain soil fungi on Metarhizium and Beauveria spp and their pathogenicity against Holotrichia consanguinea. Indian Phytopathology. 1999;52(2):196-7.

84. McKinnon AC, Glare TR, Ridgway HJ, Mendoza-Mendoza A, Holyoake A, Godsoe WK, et al. Detection of the entomopathogenic fungus Beauveria bassiana in the rhizosphere of

wound-stressed Zea mays plants. Frontiers in Microbiology. 2018;9:1161. doi:10.3389/fmicb.2018.01161
85. Bruck DJ. Fungal entomopathogens in the rhizosphere. In: Roy HE, Vega, F.E., Chandler, D., Goettel, M.S., Pell, J., Wajnberg, E., editor. The ecology of fungal entomopathogens: Springer; 2009. p. 103-12.

Supporting information

S1 Table. Estimated regression parameters, standard error, z-values, and P-value for the binomial (probit) GLMM conducted to assess proportion mortality by isolate treatment. The estimated variance for the random effect 'bioassay' is 0.016.

Figure

Click here to access/download;Figure;Fig 1.tif

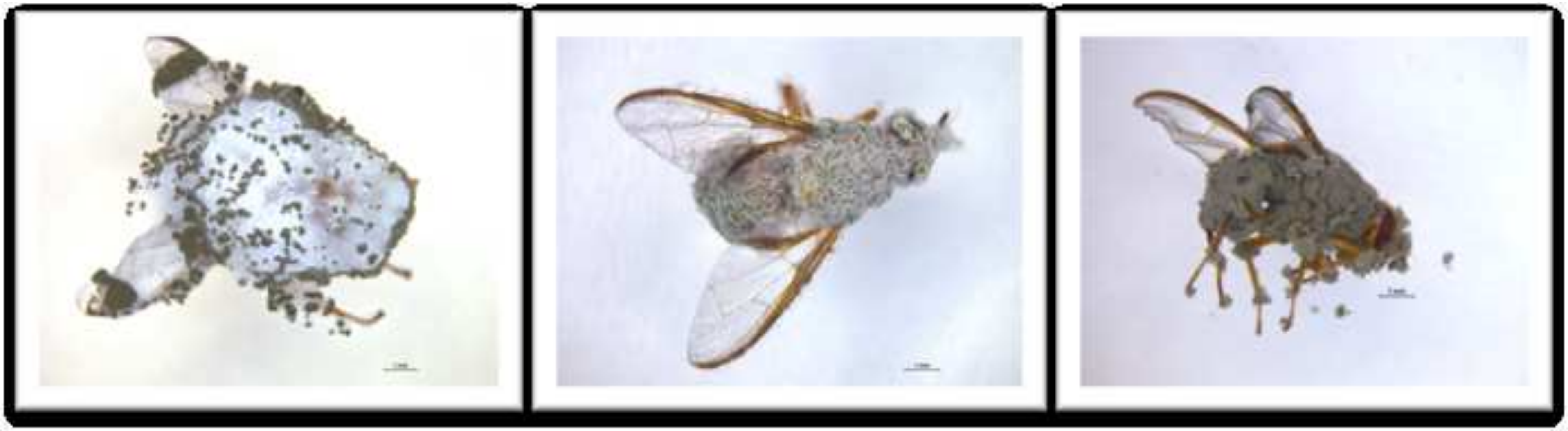

 Click here to access/download;Figure;Fig 2.tif

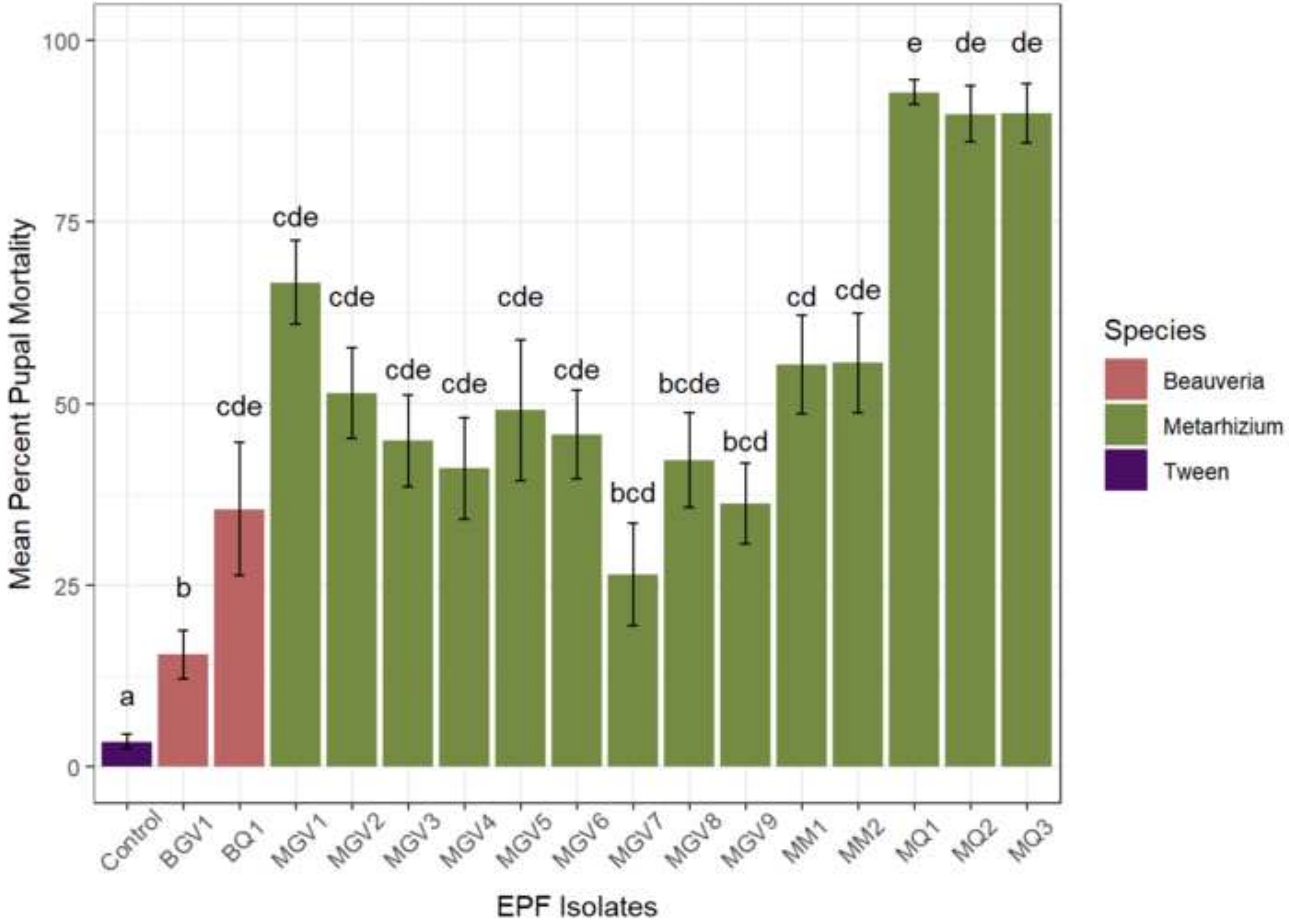

Figure

Click here to access/download;Figure;Fig 3.tif

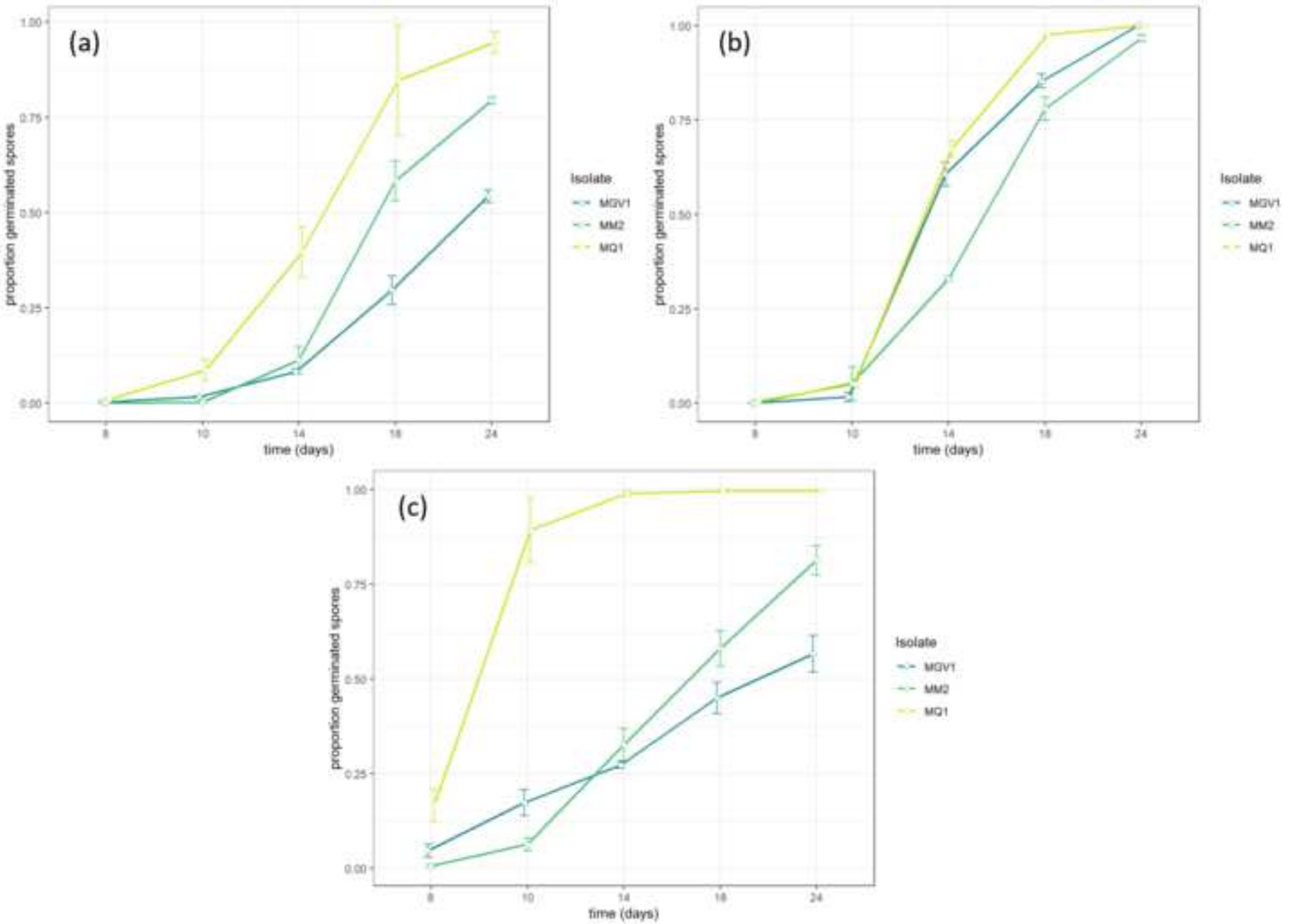

 Click here to access/download;Figure;Fig 4.tif

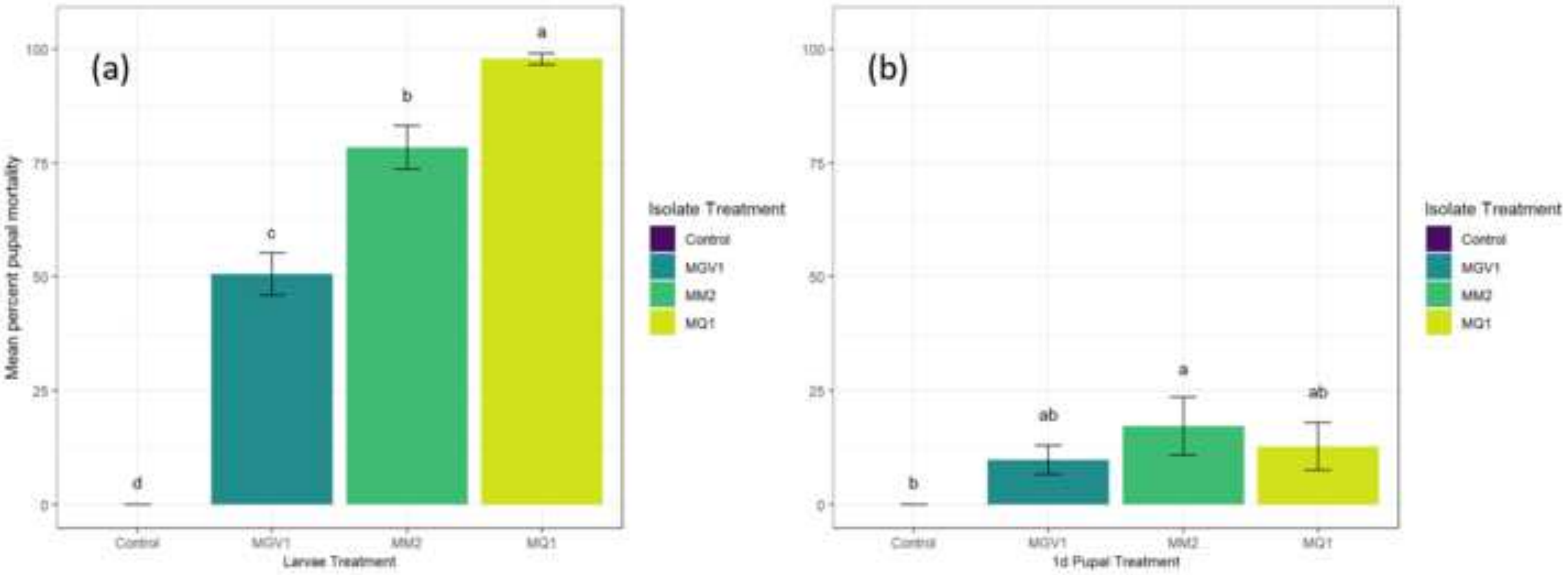

 Click here to access/download;Figure;Fig 5.tif

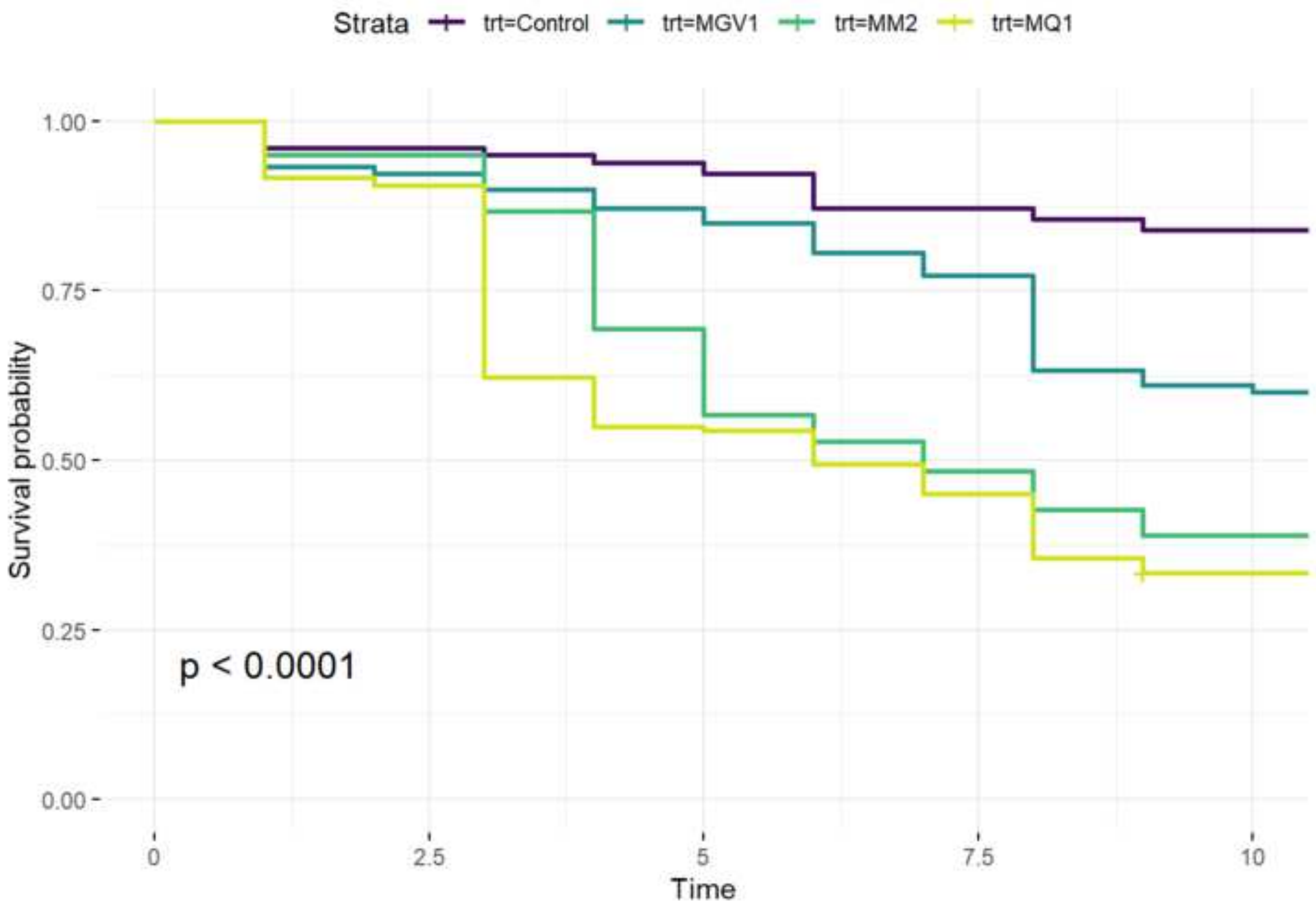

Figure

Click here to access/download;Figure;Fig 6.tif

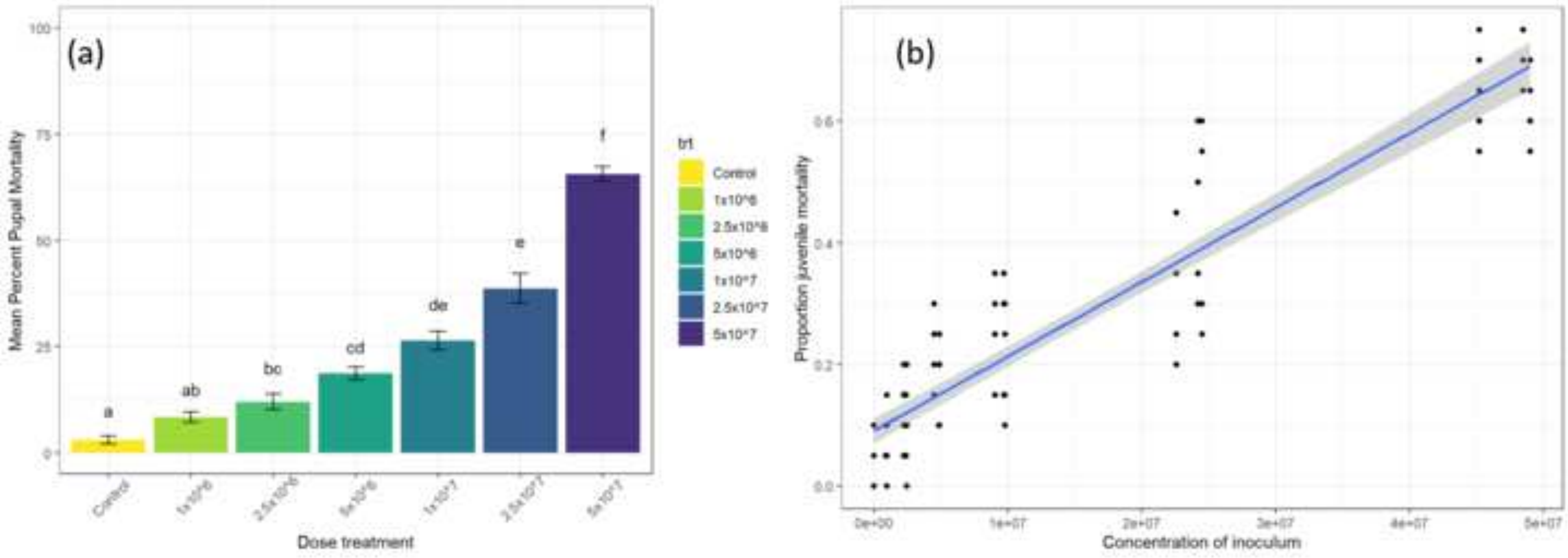

Click here to access/download
Supporting Information
S1 Table.tif
