# Supplementary material for: Metarhizium spp. isolates effective against Queensland fruit fly juvenile life stages in soil
Source: PLoS One. 2024 Jan 18;19(1):e0297341. doi: 10.1371/journal.pone.0297341 (PMC10796031; doi:10.1371/journal.pone.0297341)
Supplement: Attachment — Submitted filename: Evaluation report.docx [file pone.0297341.s005.docx]

**Review of the manuscript:**

Manuscript number: PONE-D-23-37553

The authors in the manuscript entitled “*Metarhizium* spp. isolates effective against Queensland fruit fly juvenile life stages in soil” investigate how different fungal isolates effect against the Queensland fruit fly. This study, which aims to combat an extremely harmful species, will shed light on future studies with other species.

The aim of the article is appropriate to the scope of the journal and up-to-date. The purpose and content of the text are well-designed.

Besides the corrections in PDF, the suggestions are reported below.

- The language of the article is inadequate. The article should definitely be read by a native speaker.
- In References section, there are some mistakes. All references must be corrected according to the rules established by the journal. Species names must be written in italic. Besides, journal names should be abbreviated.

**My decision is**: Major revision
