# Supplementary material for: Metarhizium spp. isolates effective against Queensland fruit fly juvenile life stages in soil
Source: PLoS One. 2024 Jan 18;19(1):e0297341. doi: 10.1371/journal.pone.0297341 (PMC10796031; doi:10.1371/journal.pone.0297341)
Supplement: Attachment — Submitted filename: Response to Reviewers.docx [file pone.0297341.s007.docx]

**Response to Reviewer	14 December 2023**

Metarhizium spp. isolates effective against Queensland fruit fly juvenile life stages in soil.

Manuscript Number: PONE-D-23-37553

**Response to reviewer 1**:

Edits have been made to address any grammatical errors where we agreed it was necessary; these changes are detailed below. Four authors are native English speakers, and we do not feel that the definite article “the” is necessary before a common name (in this case, Queensland fruit fly). We do, however, very much appreciate the thoroughness of the review provided and hope that the revised version of the manuscript is considered more than adequate with respect to the grammar and readability. The reference section was also corrected as per the requirements of the journal.

**The following comments were addressed, and changes made if necessary:**

1. Removing of the space before percentage (Lines: 38, 39, 43, 44, 109,114, 115, 126, 130, 131, 132, 138, 139, 161, 170, 179, 181, 185, 186, 188, 205, 218, 219, 239, 265, 267, 268, 346, 354, 355, 356, 358, 364, 365, 366, 367, 369, 373, 382, 383, 395, 397, 399, 400, 419, 421, 422, 462, 463, 490, 515, 517, 519, and 523).
2. We have not added an article before Queensland fruit fly. Queensland fruit fly is a common name of a species and therefore does not need an article. Comments were made by the reviewer in this regard in lines’ 29, 35, 48, 54, 58, 67, 90, 93, 101, 108, 149, 168, 222, 371, 403, 411, 414, 430, 434, 465, 470, 499, 517, 531, and 533. To keep it more consistent, articles where additionally removed before common names of other fruit fly species in line 52, 84, 418, 420, 429, 445, 453, 476, 477, and 520.
3. We made the suggested minor grammatical changes by the reviewer in the following lines:

34, 51, 60, 78, 79, 84, 87, 88, 91, 93, 96, 101, 109, 113, 114, 118, 121, 123, 124, 125, 128, 129, 131, 132, 135, 138, 139, 151, 155, 156, 161, 184, 185, 205, 209, 210, 215, 232, 233, 236, 240, 244, 245, 269, 289, 304, 309, 348, 353, 355, 360, 381, 390, 391, 393, 397, 430, 431, 437, 446, 456, 458, 459, 464, 465, 478, 481, 482, 488, 491, 493, 494, 497, 502, 510, 513, 516, 518, 520, 521, 524, and 526.

4. We paraphrased the sentence in line 354, as advised.
5. We don’t think an article in front of third-instar larvae is necessary. Comments were made by the reviewer in this regard in lines’ 36, 41, 103, 166, 167, 221, 257, 296, 297, 347, 360, 361, 363, 371, 402, 419, 435, 499, 501, and 515.
6. We have not changed the spelling of litre (Australian/ British English) to liter (American English) in line 115, because we have used Australian/Bristish English throughout. Same goes for the suggested change from centre to ‘center’ (the latter being American English) in line 198.
7. We have not hyphenated colony forming units as suggested in line 174, because it is typically written without a hyphen and uses the acronym ‘CFUs’ and not ‘C-FUs’, in much of the scientific literature.
8. Line 178. The authors felt the suggested change was unnecessary and doesn’t improve or alter the readability of the MS.
9. We decided not to hyphenate surface sterilised pupae as suggested in line 186, as it is not conventional to do this.
10. We have added the species name before isolate names for clarification (Line 299, 302, 348, 349, 360, 378, 481, 500, 501, and 513).
11. We did not make the suggested change in line 470 “Ranging from 5 to 31 days”, as this is implied by the written format and seems redundant to state.

**Response to reviewer 2:**

On behalf of the authors, we went through the comments and made changes to clarify content when suggested.

**The following comments were addressed, and changes made:**

1. We specified the larval stage tested as suggested in line 96.
2. The treatment was specified as suggested in line 129 and 135.
3. To clarify the isolates details, the caption of table 2 was edited (line 345).
4. Isolate details were clarified in line 191, 192 as well as in lines’ 348, 349, as advised.
5. We added information about two field studies. The first study was using *M. anisopliae* alone and in combination with a spinosad based bait spray against Oriental fruit fly (*B. dorsalis*) in Kenya, whereas the second study was using a combination of fungal entomopathogens (*M. anisopliae* or *B. bassiana*) and entomopathogenic nematodes (*S. carpocapsae* or *H. bacteriophora*) against peach fruit fly (*B. zonata*) and Oriental fruit fly (*B. dorsalis*) (line 529 and following). These studies suggesting that fungal isolates can be part of an effective IPM approach for field suppression of pestiferous fruit flies. Field efficacy studies of the fungal isolates have been suggested for future studies, however, the manuscript presents a complete body of work, is novel and will add value to field of research.
