# Supplementary material for: Metarhizium spp. isolates effective against Queensland fruit fly juvenile life stages in soil
Source: PLoS One. 2024 Jan 18;19(1):e0297341. doi: 10.1371/journal.pone.0297341 (PMC10796031; doi:10.1371/journal.pone.0297341)
Supplement: Attachment — Submitted filename: Evaluation report-2.docx [file pone.0297341.s008.docx]

**Review of the manuscript:**

Manuscript number: PONE-D-23-37553

When I reviewed the PDF, I noticed that the authors had made all the necessary corrections. The article can be printed with this version.

**My decision is:** Accept
